# Precision Dosing for Pediatric Growth Hormone Deficiency With Daily and Weekly Growth Hormone

**DOI:** 10.1002/mco2.70945

**Published:** 2026-08-24

**Authors:** Wei Wu, Chun‐Xiu Gong, Jie Mei, Ling Hou, Yan Liang, Yan‐qin Ying, Feng Ye, Ge‐Hang Ju, Hui‐Xiang Tian, Nan Yang, Yun Qin, Dongsheng Ouyang, Xiaoping Luo

**Affiliations:** ^1^ Department of Pediatrics Tongji Hospital, Tongji Medical College, Huazhong University of Science and Technology; Hubei Provincial Key Laboratory of Pediatric Genetic Metabolic and Endocrine Rare Diseases; Hubei Provincial Clinical Research Center for Children's Growth and Development and Metabolic Diseases; State Key Laboratory for Diagnosis and Treatment of Severe Zoonotic Infectious Disease Wuhan China; ^2^ Department of Endocrinology Genetics and Metabolism Beijing Children's Hospital, Capital Medical University, National Centre for Children's Health Beijing China; ^3^ Tianfu Jincheng Laboratory Chengdu China; ^4^ Institute of Big Data, Central South University Changsha China; ^5^ Department of Clinical Pharmacology Xiangya Hospital, Central South University Changsha China; ^6^ Center of Clinical Pharmacology Third Xiangya Hospital, Central South University Changsha China; ^7^ Department of Radiology West China Hospital, Sichuan University Chengdu China

**Keywords:** dose switching, growth hormone deficiency, PEG‐rhGH, PK/PD model, rhGH

## Abstract

Pediatric growth hormone deficiency (GHD) typically requires daily injections of recombinant human growth hormone (rhGH). Long‐acting PEGylated recombinant human growth hormone (PEG‐rhGH) formulations have been developed to reduce injection frequency and improve adherence and quality of life. However, guidance on switching between short‐acting and long‐acting regimens during treatment and biomarker‐driven precision dose‐conversion strategies remains lacking in real‐world clinical practice. Therefore, this study analyzed data from two populations comprising healthy adult male subjects (*n* = 39) and pediatric patients with GHD (*n* = 408). The analysis used population pharmacokinetic (PopPK) and population pharmacokinetic/pharmacodynamic (PopPK/PD) models. The models showed adequate fit and external predictability. Biomarker‐clinical growth endpoint relationship analysis linked changes in insulin‐like growth factor‐1 standard deviation score (IGF‐1 SDS) to growth outcomes, and simulations assessed subgroup responses, missed‐dose scenarios, and IGF‐1 SDS‐guided switching. Simulations enabled subgroup‐specific efficacy projections. Missed doses caused a greater loss of benefit with rhGH than with PEG‐rhGH. Switching simulations supported IGF‐1 SDS‐guided conversion within accepted safety limits. The integrated framework provides concise, clinically interpretable rules for individualized titration, adherence‐aware care, and safer switching in pediatric GHD. This study supports precision dosing, missed‐dose management, and switching between daily rhGH and weekly PEG‐rhGH in pediatric GHD.

## Graphical abstract

• This study supports precision dosing and switching between daily rhGH and weekly PEG‐rhGH in pediatric GHD.

• Missed doses reduce treatment benefit more with daily rhGH than with weekly PEG‐rhGH.

• The findings provide guidance for growth hormone dosing and monitoring in pediatric GHD.

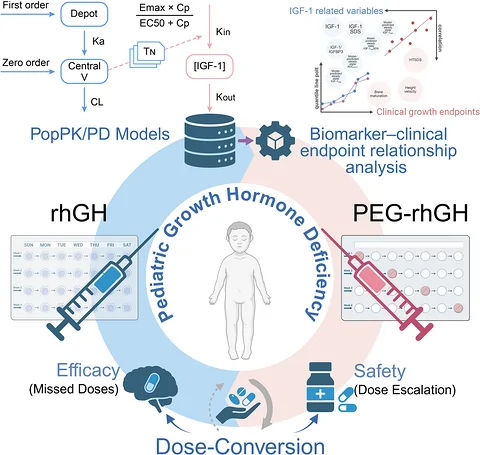

## 1 Introduction

Growth hormone deficiency (GHD) in children is an endocrine disorder caused by insufficient secretion of growth hormone (GH) from the anterior pituitary, primarily manifesting as short stature [1]. Currently, short‐acting recombinant human growth hormone (rhGH) replacement therapy is the standard treatment for GHD [2], requiring daily injections for children with GHD. However, in the real world, adherence is often suboptimal, and missed injections are common, leading to significant fluctuations in insulin‐like growth factor‐1 (IGF‐1) levels, unstable efficacy, and long‐term adverse outcomes [3, 4].

To address this, long‐acting PEGylated recombinant human growth hormone (PEG‐rhGH) formulations has been developed, aiming to reduce the injection frequency, alleviate the psychological and caregiving burden on children and their families, and improve patient adherence and quality of life [5, 6]. Several randomized controlled trials and meta‐analyses have shown that long‐acting growth hormone provides efficacy comparable to that of short‐acting growth hormone in promoting height growth, annual growth velocity, and other measures [7], with similar safety profiles and no significant differences in the incidence of adverse events [8]. However, clear and practical recommendations for switching between short‐acting and long‐acting formulations remain lacking, and clinical practice still lacks biomarker‐based strategies for precise dose conversion. Specifically, there is a lack of guidance on the safe and effective window for switching between long‐acting and short‐acting formulations, as well as on dose stratification for different age and weight groups, and the quantification of IGF‐1 target window management.

In routine care, daily rhGH dosing in pediatric GHD is individualized mainly by growth response, and IGF‐1 is used primarily for safety/adherence monitoring with dose reduction recommended when IGF‐1 is persistently above the age‐ and pubertal stage appropriate range [9]. Although model‐informed strategies for IGF‐1 guided dosing in once‐weekly long‐acting GH have been proposed, including standardized post‐dose sampling and estimation of weekly mean IGF‐1, the evidence base is still insufficient to support robust, individualized titration and switching decisions across patients [10, 11]. Pharmacometrics can move beyond IGF‐1 only switching by building population pharmacokinetic/pharmacodynamic (PopPK/PD) models to describe exposure and the full IGF‐1 time course, then linking predicted IGF‐1 dynamics to growth outcomes in an exposure–response framework. Our prior work characterized the relationship between GH and IGF‐1 in children with GHD receiving PEG‐rhGH [12], providing a foundation for individualized titration, dose‐conversion recommendations, switching windows, and missed‐dose management.

Therefore, this study built an integrated population PK/PD and exposure–response framework in children with GHD to connect drug exposure to the full IGF‐1 time course and then link IGF‐1 dynamics to clinical growth outcomes. Using this unified model, we compared daily rhGH and weekly PEG‐rhGH, evaluated individualized dose adjustment, and tested practical switching approaches between formulations. We also quantified the impact of missed, delayed, or off‐label dosing on IGF‐1 trajectories and predicted growth. The intended clinical impact is a simple, model‐informed set of titration and conversion rules that supports safer switching and more consistent growth during long‐term therapy.

## 2 Results

### 2.1 PopPK Analysis

The PopPK model for rhGH was developed in this study, while the PEG‐rhGH PopPK model was based on a previously established model [12]. The rhGH model was developed using 504 pharmacokinetic (PK) observations from 21 participants. The previously established PEG‐rhGH PopPK model was used to support subsequent simulations and comparisons. Details of the supporting clinical studies are provided in Tables S1 and S2.

**FIGURE 1:**
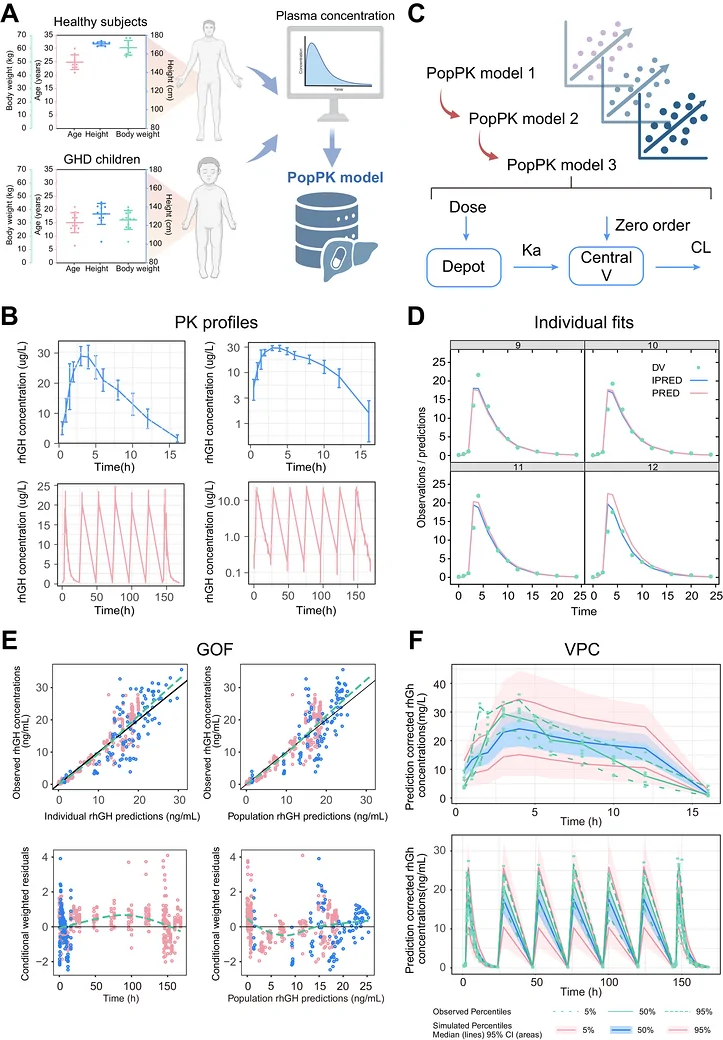
Development of population pharmacokinetic models for rhGH in healthy participants and GHD children. (A) Overall workflow for population pharmacokinetic analysis. (B) Pharmacokinetic concentration‐time profiles following subcutaneous rhGH administration in healthy participants and children with GHD. (C) Development and selection of the base PopPK model for rhGH. (D) Individual fits for the rhGH PopPK model. (E) Goodness‐of‐fit plots for the rhGH PopPK model, where blue circles represent healthy participants and red circles represent participants with GHD. (F) Visual predictive check plot for the rhGH PopPK model. In the VPC plots, green points and dashed/solid lines represent the observed percentiles, solid colored lines represent the simulated percentiles, and shaded areas represent the corresponding 95% simulation intervals. GHD, growth hormone deficiency; PopPK, population pharmacokinetic; rhGH, recombinant human growth hormone; VPC, visual predictive check.

Figure 1A outlines the general workflow of the PopPK analysis. Mean and individual concentration‐time profiles of rhGH for healthy participants and children with GHD are presented in Figure 1B and Figures S1 and S2. Dose‐normalized exposure was found to be higher in GHD children compared to healthy participants (Figure S3). Following the screening of candidate base models (Figure 1C), the PopPK analysis identified a two‐compartment model with mixed zero‐ and first‐order absorption as optimal for describing pharmacokinetics of rhGH in both populations, with below the limit of quantification (BLQ) data handled using the M1 method. The rhGH model diagnostics, including individual fits, goodness‐of‐fit (GOF), and visual predictive checks (VPC) results, are presented in Figure 1D–F, and the final PopPK parameter estimates are provided in Table S3. In the GOF plots, the loess trend line closely followed the line of identity, and conditional weighted residuals (CWRES) were symmetrically distributed around zero and largely within ±4. In the VPC plots, the observed fifth, 50th, and 95th percentiles were generally covered by the 95% prediction intervals of the simulations. Overall, these results support that the developed rhGH PopPK models adequately capture the PK characteristics of rhGH in both healthy participants and pediatric GHD patients.

### 2.2 PopPK/PD Analysis

**FIGURE 2:**
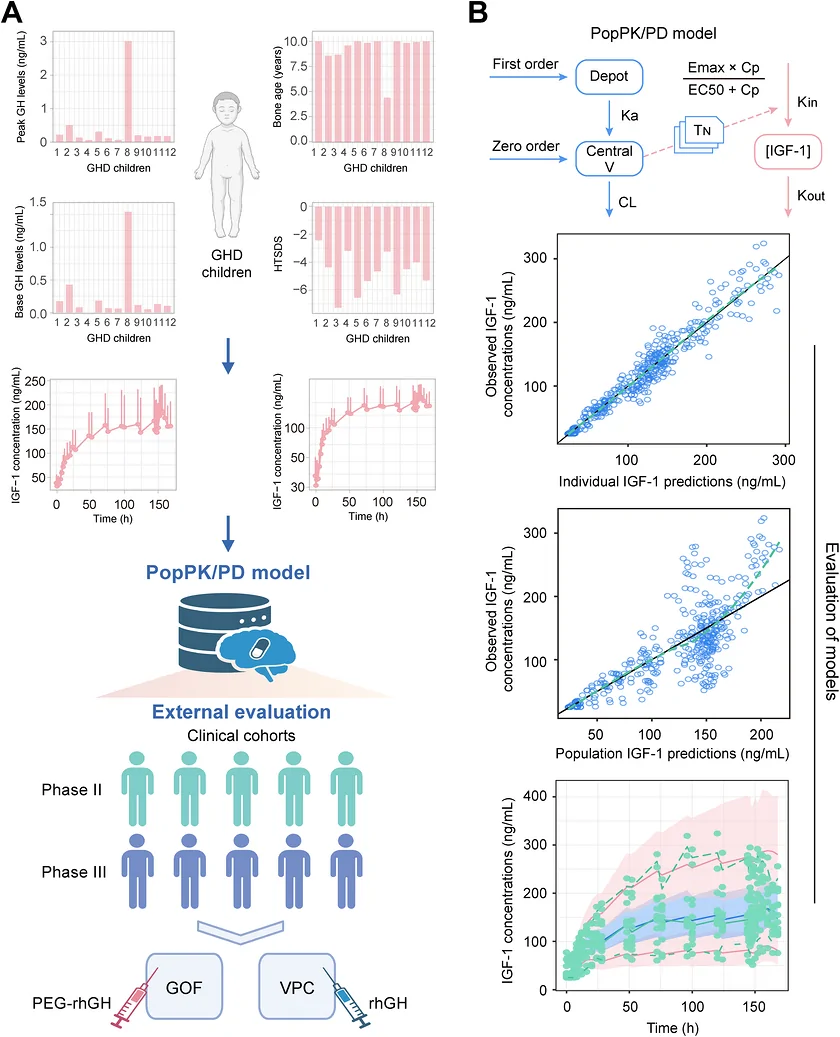
Development of the rhGH PopPK/PD model in children with GHD. (A) Overall schematic workflow of PopPK/PD modeling for rhGH. (B) Structural diagram and diagnostic evaluation plots of the rhGH PopPK/PD model. GHD, growth hormone deficiency; PopPK/PD, population pharmacokinetic/pharmacodynamic; rhGH, recombinant human growth hormone.

Figure 2A outlines the general workflow of the PopPK/PD analysis. Individual IGF‐1 profiles of GHD are plotted in Figure S4. Exploratory analysis indicated that after subcutaneous administration of rhGH, IGF‐1 levels exhibited a temporal trend similar to but delayed relative to plasma drug concentrations (Figures S5–S7). Accordingly, an indirect response model was selected as the structural base for the PopPK/PD model (Figures S8–S10, key building steps summarized in Table S4). Following covariate selection (Figure S11), baseline IGF‐1 (BIGF‐1) was identified as a statistically significant covariate on the baseline effect, reducing the objective function value by 28.44.

The base and final PD parameter estimates are presented in Table S5. The final model showed improved goodness‐of‐fit performance compared with the base model and reduced the inter‑individual variability of baseline effect to negligible levels, indicating that BIGF‐1 explained a substantial portion of the between‐subject variation. Figure 2B and Figure S12 illustrate the final PopPK/PD model structure and overall model fit, including diagnostic evaluation of the final rhGH PopPK/PD model.

### 2.3 External Evaluation of the PopPK/PD Models

**FIGURE 3:**
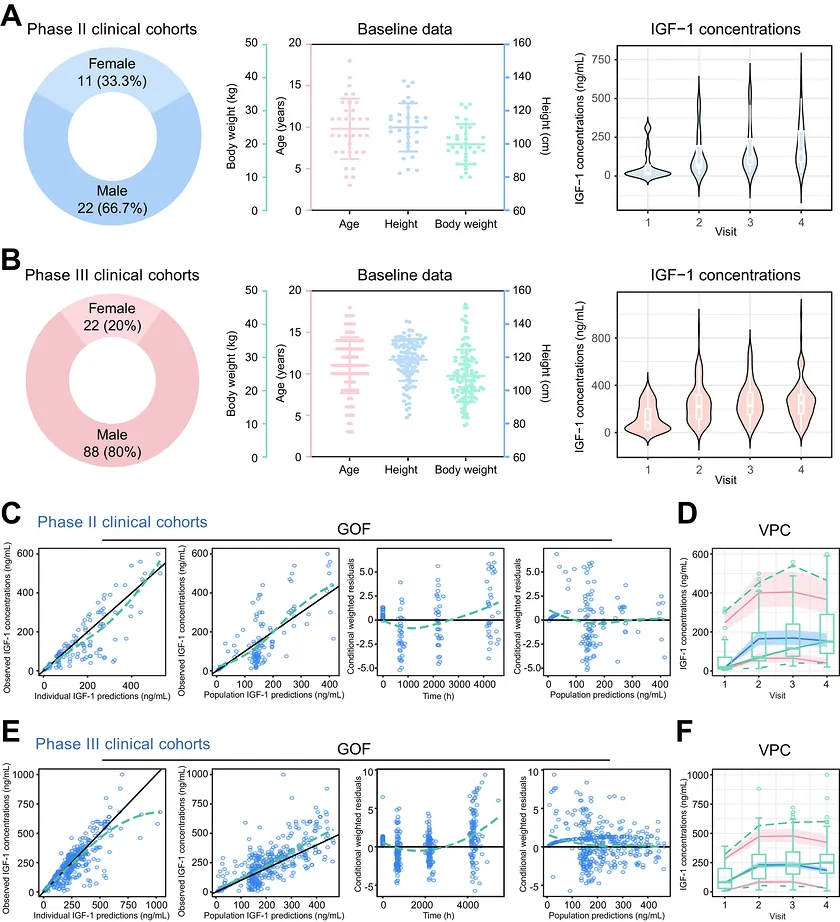
External evaluation of the rhGH PopPK/PD model using Phase II and Phase III clinical cohorts. (A) Demographic characteristics, baseline data, and IGF‐1 concentration distributions in the Phase II clinical cohort. (B) Demographic characteristics, baseline data, and IGF‐1 concentration distributions in the Phase III clinical cohort. (C) Goodness‐of‐fit plots for external evaluation in the Phase II clinical cohort. (D) Visual predictive check plot for external evaluation in the Phase II clinical cohort. (E) Goodness‐of‐fit plots for external evaluation in the Phase III clinical cohort. (F) Visual predictive check plot for external evaluation in the Phase III clinical cohort. IGF‐1, insulin‐like growth factor‐1; PopPK/PD, population pharmacokinetic/pharmacodynamic; rhGH, recombinant human growth hormone.

External evaluation results using Phase II and Phase III clinical data for rhGH are presented in Figure 3. Figure 3A,B summarizes the demographic characteristics, baseline data, and IGF‐1 concentration distributions of the Phase II and Phase III validation cohorts, respectively. Detailed characteristics of the external validation cohorts are provided in Table S6. Figure 3C,D presents the GOF and VPC results for the Phase II validation cohort, while Figure 3E,F presents the corresponding results for the Phase III validation cohort. Good agreement was observed between observed and predicted values. Similarly, the external evaluation for PEG‐rhGH also demonstrated acceptable model fit, as shown in Figures S13 and S14. These results support the adequacy of the final PopPK/PD models in describing the IGF‐1 response in the studied population.

### 2.4 Biomarker‑Clinical Growth Endpoint Relationship Analysis

**FIGURE 4:**
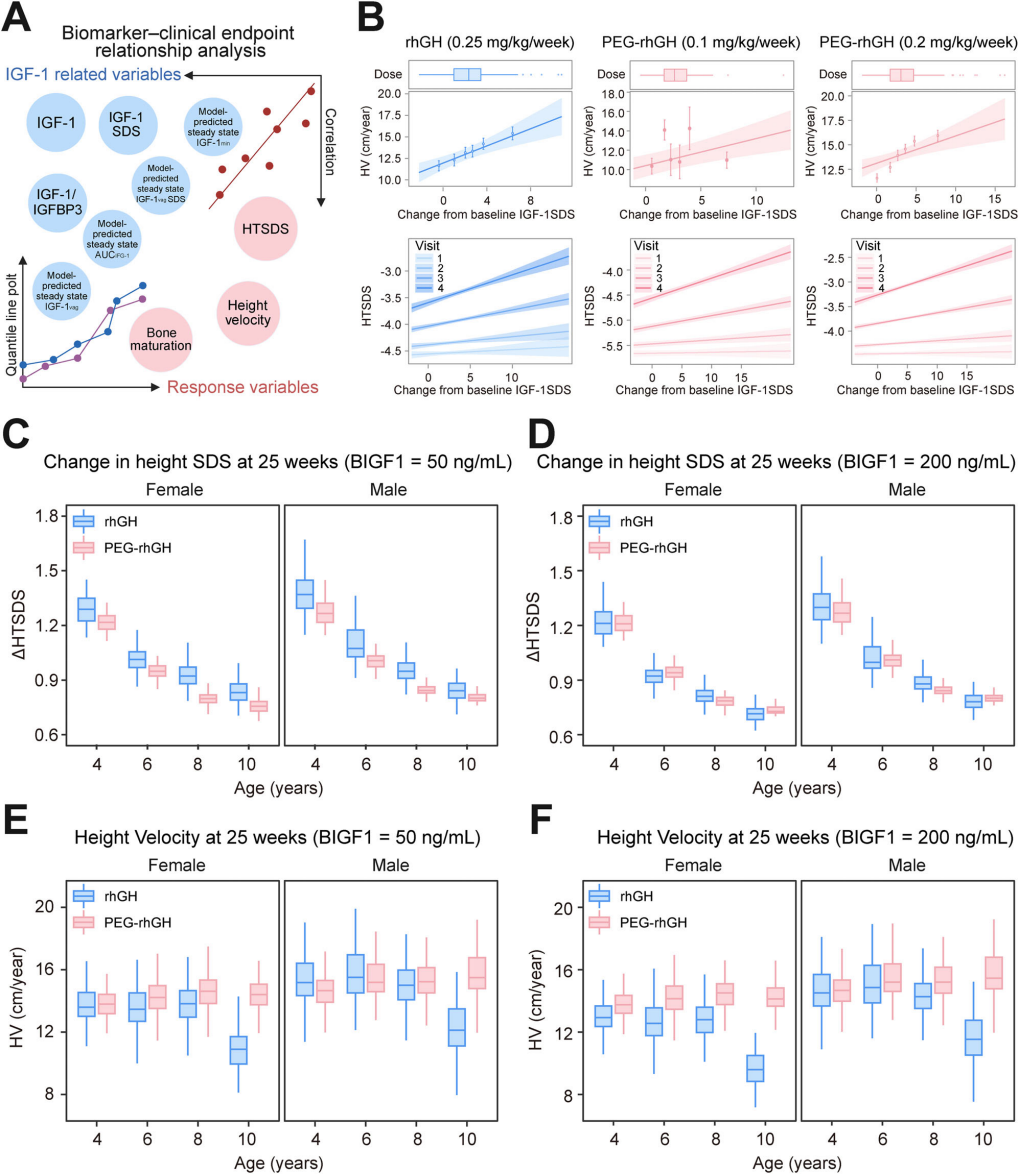
Development and application of biomarker‑clinical growth endpoint relationship models for rhGH and PEG‐rhGH. (A) Exploratory analysis of the relationship between IGF‐1‐related variables and clinical growth endpoints. Blue circles indicate IGF‐1‐related biomarker variables, and pink circles indicate clinical growth endpoints. (B) Schematic of the established biomarker‑clinical growth endpoint relationship models for daily rhGH and weekly PEG‐rhGH. In the upper panels, the x‐axis represents change from baseline in IGF‐1 SDS, and the y‐axis represents height velocity. In the lower panels, the x‐axis represents change from baseline in IGF‐1 SDS, and the y‐axis represents HTSDS across visits. (C) Model‐simulated changes in HTSDS at 25 weeks following administration of rhGH and PEG‐rhGH (BIGF‐1 = 50 ng/mL). (D) Model‐simulated changes in HTSDS at 25 weeks following administration of rhGH and PEG‐rhGH (BIGF‐1 = 200 ng/mL). (E) Model‐simulated changes in HV at 25 weeks following administration of rhGH and PEG‐rhGH (BIGF‐1 = 50 ng/mL). (F) Model‐simulated changes in HV at 25 weeks following administration of rhGH and PEG‐rhGH (BIGF‐1 = 200 ng/mL). Panels C–F present model‐based projections stratified by sex and age, rather than directly observed clinical outcomes. BIGF‐1, baseline insulin‐like growth factor‐1; HTSDS, height standard deviation score; HV, height velocity; IGF‐1, insulin‐like growth factor‐1; IGF‐1 SDS, insulin‐like growth factor‐1 standard deviation score; PEG‐rhGH, PEGylated recombinant human growth hormone; rhGH, recombinant human growth hormone.

Figure 4 illustrates how changes in IGF‐1‐related biomarkers were translated into expected clinical growth endpoints, including height standard deviation score (HTSDS) and height velocity (HV), through the biomarker–clinical growth endpoint relationship models. Based on exploratory and correlation analyses (Figure 4A, Figures S15–S18), linear mixed‐effects models for HTSDS and IGF‑1 SDS change from baseline, and linear models for HV and IGF‑1SDS change from baseline, were developed separately for rhGH and PEG‑rhGH using Phase II/III data in children with GHD. In summary, both short‐acting and long‐acting GH treatments showed a significant positive interaction between baseline IGF‐1 SDS and time on height SDS progression (*p* < 0.0001 for both models). Change from baseline IGF‐1 SDS was independently associated with growth velocity in both treatment groups (*p* < 0.01). Age splines, bone age, mid‐parental height, and sex were also significant predictors in the respective models. Detailed equations and complete parameter estimates are provided in Tables S7–S10. The results, summarized in Figure 4B, show that error bars for both rhGH and PEG‑rhGH largely fall within their respective confidence intervals, supporting the robustness of the established biomarker‑clinical relationship models.

Based on the simulation framework and real‐world GHD cohort characteristics described in Table S11, expected changes in HV and HTSDS after 25 weeks were projected for children aged 4, 6, 8, and 10 years. Two BIGF‐1 levels (50 and 200 ng/mL) were evaluated to assess the impact of this key covariate on treatment response.

Simulation results are summarized in Figure 4C–F. As shown in Figure 4C,D, the improvement in HTSDS declines with increasing age. Figure 4E,F suggests that the effect of PEG‑rhGH on HV remains consistent across age groups, whereas the effect of rhGH appears attenuated in 10‑year‑olds compared to younger children. This difference may reflect reduced rhGH responsiveness in older children, although the interpretation should also account for the relatively smaller sample size in the 10‑year‑old subgroup.

### 2.5 Impact of Missed Doses

**FIGURE 5:**
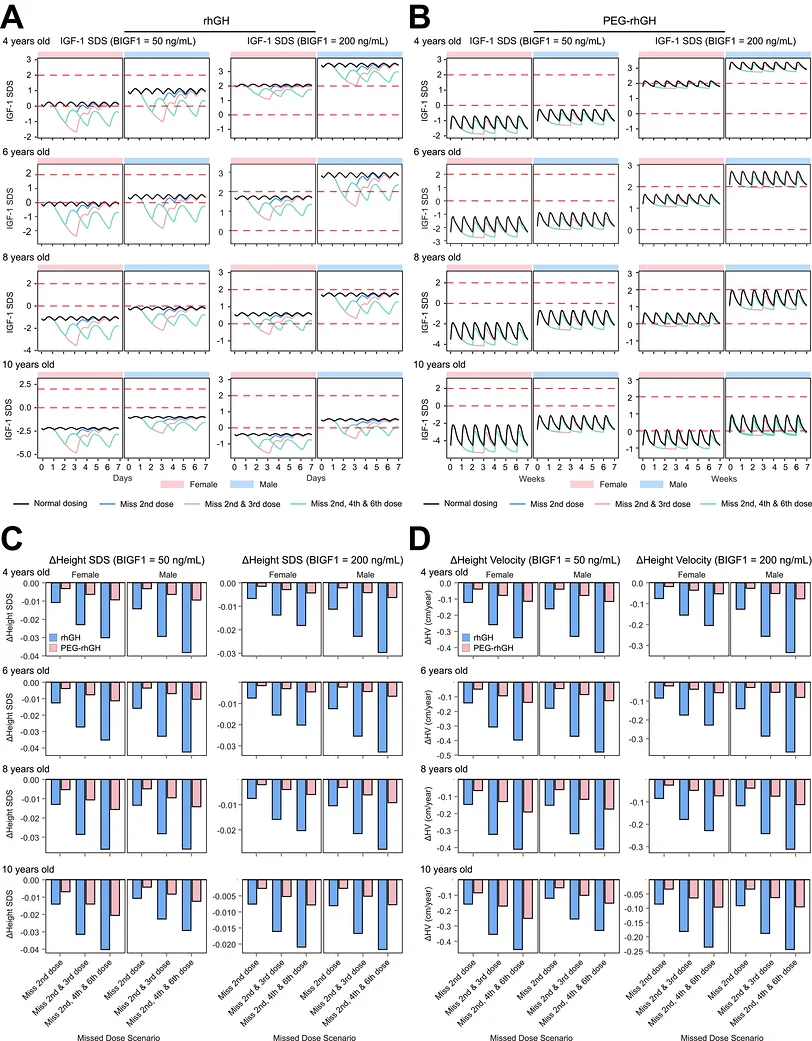
Impact of missed doses on IGF‐1 SDS, HTSDS, and HV for rhGH and PEG‐rhGH. (A) Simulated changes in IGF‐1 SDS after missed injections of daily rhGH. Different colored lines represent different missed‐dose scenarios, and dashed horizontal lines indicate reference thresholds for IGF‐1 SDS. (B) Simulated changes in IGF‐1 SDS after missed injections of weekly PEG‐rhGH. Different colored lines represent different missed‐dose scenarios, and dashed horizontal lines indicate reference thresholds for IGF‐1 SDS. (C) Simulated changes in HTSDS following missed doses of daily rhGH and weekly PEG‐rhGH. Bars represent model‐predicted differences from the corresponding full‐adherence scenario across age, sex, and BIGF‐1 strata. (D) Simulated changes in annualized HV following missed doses of daily rhGH and weekly PEG‐rhGH. Bars represent model‐predicted differences from the corresponding full‐adherence scenario across age, sex, and BIGF‐1 strata. These simulations represent model‐based projections of non‐adherence scenarios rather than directly observed clinical outcomes. BIGF‐1, baseline insulin‐like growth factor‐1; HTSDS, height standard deviation score; HV, height velocity; IGF‐1 SDS, insulin‐like growth factor‐1 standard deviation score; PEG‐rhGH, PEGylated recombinant human growth hormone; rhGH, recombinant human growth hormone.

Figure 5 evaluates the impact of missed doses on IGF‐1 SDS, HTSDS, and HV under various missed‐dose scenarios for rhGH and PEG‐rhGH. The impact of missed doses was significantly greater for rhGH compared to PEG‐rhGH. As the number of missed doses increased, the effect on IGF‐1 SDS became more pronounced. For rhGH, missing three doses prevented IGF‐1 SDS recovery to normal levels within seven dosing occasions, whereas PEG‐rhGH exhibited smaller IGF‐1 SDS fluctuations and greater tolerance to non‐adherence (Figure 5A,B). Across all age groups and regardless of BIGF‐1 levels, the effects of missed doses on HTSDS and HV were greater for rhGH than for PEG‐rhGH under equivalent conditions (Figure 5C,D).

### 2.6 Dose Conversion and Escalation Exploration

**FIGURE 6:**
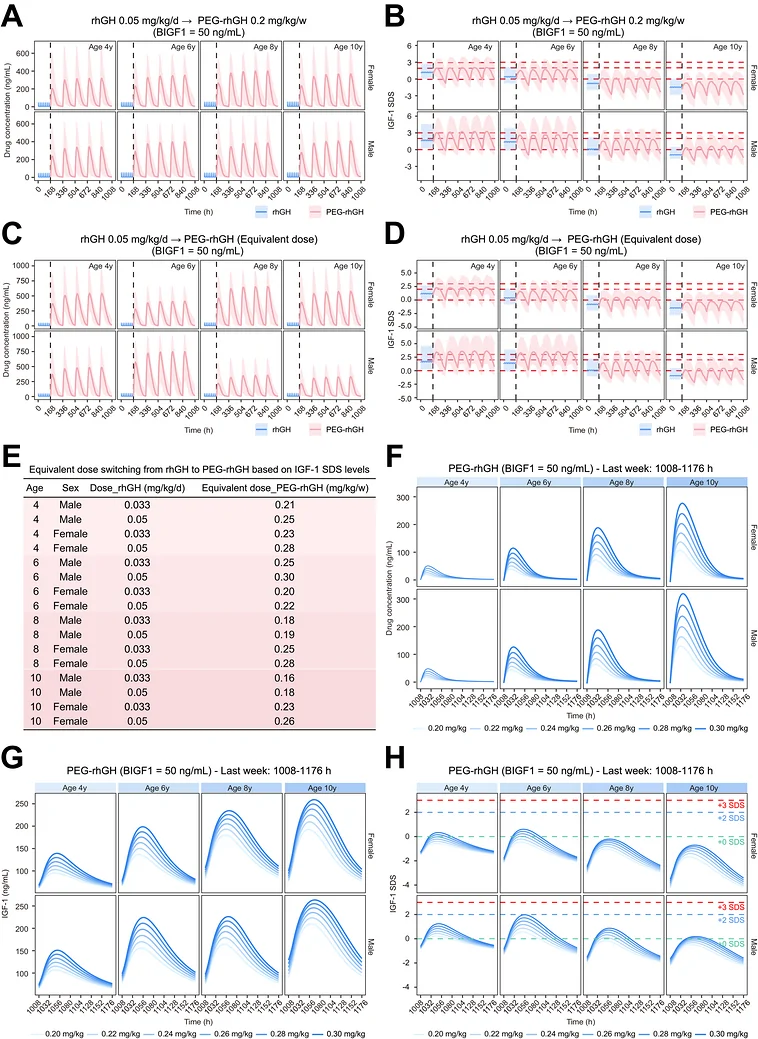
Dose‐equivalence exploration and safety assessment for switching from rhGH to PEG‐rhGH in children with GHD. (A) Simulated serum drug concentration profiles following the switch from daily rhGH (0.05 mg/kg/day) to weekly PEG‐rhGH (0.2 mg/kg/week). (B) Simulated IGF‐1 SDS profiles following the switch from daily rhGH (0.05 mg/kg/day) to weekly PEG‐rhGH (0.2 mg/kg/week). (C) Simulated serum drug concentration profiles after switching from daily rhGH (0.05 mg/kg/day) to equivalent weekly doses of PEG‐rhGH. (D) Simulated IGF‐1 SDS profiles after switching from daily rhGH (0.05 mg/kg/day) to equivalent weekly doses of PEG‐rhGH. In Panels A–D, solid lines represent model‐predicted median profiles, and shaded areas represent the corresponding prediction intervals. Panels A and B represent switching to the labeled PEG‐rhGH dose, whereas Panels C and D represent switching to model‐derived equivalent PEG‐rhGH doses targeting comparable IGF‐1 SDS exposure. (E) Equivalent dose conversion table for switching from daily rhGH (0.033 and 0.05 mg/kg/day) to weekly PEG‐rhGH. (F) Simulated serum drug concentration profiles across multiple dose levels of PEG‐rhGH. (G) Simulated IGF‐1 concentration profiles across multiple dose levels of PEG‐rhGH. (H) Simulated IGF‐1 SDS profiles across multiple dose levels of PEG‐rhGH. In Panels F–H, different blue lines indicate different PEG‐rhGH dose levels. Dashed horizontal lines in Panel H indicate reference thresholds of 0, +2, and +3 SDS. GHD, growth hormone deficiency; IGF‐1, insulin‐like growth factor‐1; IGF‐1 SDS, insulin‐like growth factor‐1 standard deviation score; PEG‐rhGH, PEGylated recombinant human growth hormone; rhGH, recombinant human growth hormone.

Figure 6 illustrates dose switching from daily rhGH to weekly PEG‐rhGH to evaluate whether the proposed equivalent doses maintained IGF‐1 SDS within clinically acceptable ranges. The short elimination half‐life of daily rhGH ensures that residual concentrations are negligible at switching and do not affect PEG‐rhGH pharmacokinetics. Figure 6A–D presents simulated pharmacokinetic and IGF‐1 SDS profiles during transition from rhGH (0.05 mg/kg/day) to PEG‐rhGH at either the labeled dose (0.2 mg/kg/week) or a model‐derived equivalent dose. As shown in Figure 6E, equivalent weekly PEG‐rhGH doses required to match common daily rhGH regimens (0.033–0.05 mg/kg/day) ranged from 0.16 to 0.30 mg/kg/week. Results for other BIGF‐1 levels and comprehensive conversion tables are provided in Figures S19 and S20 and Table S12. Exploratory analysis of the reverse switch (from PEG‐rhGH to rhGH) suggested challenges in achieving complete IGF‐1 SDS equivalence in some subgroups (Figures S21–S26), primarily due to the pharmacokinetic tail of PEG‐rhGH and age‐dependent IGF‐1 SDS reference ranges. As this scenario is less common and often involves pubertal patients beyond this study's focus, specific dose recommendations are not provided, and clinical monitoring is advised.

To evaluate the safety profile of the identified equivalent dose range, PK/PD simulations were performed with dose escalation. Within the proposed dose range (0.16–0.30 mg/kg/week), IGF‐1 SDS remained below the +3 SDS warning threshold across all BIGF‐1 levels examined (Figure 6F–H, Figures S27 and S28). For typical patients (BIGF‐1 = 50 ng/mL), IGF‐1 SDS remained within the clinically acceptable range (≤+2 SDS) throughout the proposed dose range, with the +3 SDS threshold not reached until doses approached 0.40 mg/kg/week, indicating additional safety margin (Figures S29–S31). These findings support the safety of the proposed equivalent dose range and identify BIGF‐1 concentration as a key determinant of IGF‐1 SDS response. For PEG‐rhGH, the relationship between single‐day IGF‐1 SDS and weekly average IGF‐1 SDS at steady state was further evaluated to support practical IGF‐1 monitoring during dose conversion and adjustment. Day 5 after PEG‐rhGH administration showed the smallest deviation from weekly average IGF‐1 SDS in the simulation analysis, and the corresponding conversion equations and prediction intervals are provided in Figure S32 and Table S13.

## 3 Discussion

The pathological cause of GHD is a dysfunction in the hypothalamic‐pituitary‐IGF‐1 “growth axis” [13]. Some studies have reported mutations in GH1, GHRHR, as well as abnormalities in pituitary development [14, 15], all of which ultimately result in insufficient GH secretion, leading to a reduction in the liver's synthesis of IGF‐1 [16], which then affects the growth and metabolism of bones [17], cartilage, and other body tissues [18]. Currently, hormonal assessment based on body fluid analysis is an important part of the clinical evaluation of patients with GHD [19, 20, 21]. Additionally, IGF‐1 and insulin‐like growth factor‐binding protein 3 (IGFBP‐3) levels can serve as auxiliary indicators [22]. Currently, growth hormone supplementation strategies have become the foundational and sole pharmacological treatment for GHD [23]. Several long‐acting growth hormone medications have also been developed [24], including Somapacitan and Lonapegsomatropin [25, 26], primarily aimed at reducing injection frequency to improve adherence [27]. Clinical trials have demonstrated low immunogenicity and favorable safety profiles, and these formulations are increasingly used internationally [28, 29].

Despite proven efficacy, both daily rhGH and long‐acting GH face implementation barriers that limit “IGF‐1‐only” decision‐making. In particular, daily injections are also associated with suboptimal adherence and discontinuation, which may compromise growth outcomes and complicate the interpretation of a single IGF‐1 value [3, 30]. In addition, long‐term safety remains under active surveillance, with observational data reporting signals for selected outcomes (e.g., mortality, cardiovascular events, and neoplasia) that warrant individualized risk–benefit assessment [31, 32, 33, 34]. Therefore, more precise dosing guidance should take into account the overall clinical context, including BIGF‐1 deficiency and early on‐treatment IGF‐1 response patterns [35, 36] and possible pharmacogenetic implementation [37].

Using pharmacometrics approaches, this study established and validated population PK/PD models for both rhGH and PEG‐rhGH in children with GHD using multi‐cohort clinical trial data. Weight and BIGF‐1 levels were identified as significant covariates affecting PK/PD performance. Furthermore, the correlation between IGF‐1‐related indicators and clinical efficacy endpoints (HV, HTSDS, and bone maturation) was characterized, establishing the foundation for subsequent analysis. Biomarker‐clinical analysis revealed that ΔIGF‐1 SDS was strongly positively correlated with HV, whereas for HTSDS, time exhibited a significant main effect and a significant interaction with ΔIGF‐1 SDS. Age was negatively correlated, while mid‐parental height was positively correlated. The results demonstrated that missed rhGH doses produced greater reductions in IGF‐1 SDS and growth parameters than missed PEG‐rhGH doses, attributable to the shorter half‐life of rhGH resulting in complete loss of drug exposure for each missed injection. Additionally, patients with lower BIGF‐1 levels exhibited greater sensitivity to missed doses. These findings suggest that long‐acting PEG‐rhGH formulations may offer greater resilience to imperfect adherence, which is particularly relevant in pediatric populations where maintaining consistent daily injections can be challenging. However, the PK/PD modeling population in this study was restricted to children with GHD and did not include children with idiopathic short stature (ISS) or pubertal children with GHD. Differences in underlying disease mechanisms and developmental stages may lead to differences in PD responses. Therefore, the findings of this study require further large‐scale validation using real‐world data to confirm and refine the proposed models and dosing strategies.

Based on simulation analyses, equivalent dosing ranges were derived for switching from rhGH to PEG‐rhGH using IGF‐1 SDS as the therapeutic target. For dose‐switching simulations, BIGF‐1 levels of 50, 100, and 200 ng/mL were evaluated to characterize dose equivalence across the range of baseline IGF‐1 concentrations considered in the simulations and to support clinically applicable dose‐switching recommendations. The proposed equivalent dose range provides guidance for clinical dose conversion, allowing physicians to select appropriate initial PEG‐rhGH doses based on prior rhGH regimens. Safety simulations confirmed acceptable margins within the proposed dose range, supporting individualized dose optimization guided by BIGF‐1 monitoring. In this study, daily rhGH preparations that meet established standards for specific activity and purity were considered pharmacokinetically and pharmacodynamically comparable and therefore suitable for conversion to U‐shaped PEG‐rhGH using the proposed switching model. While this model is not directly applicable to non‐U‑shaped PEG‐rhGH due to their different structural modifications, the overall study design and PK/PD assessment strategy can nonetheless inform the development of analogous transition approaches for other long‐acting formulations.

Overall, using multi‐cohort data and IGF‐1 as the central biomarker, this study established and validated population PK/PD and biomarker‑clinical relationship models for long‐acting and short‐acting growth hormones in children with GHD. This study elucidated the impact of adherence patterns and missed injections on therapeutic outcomes, and established practical frameworks for dose conversion when switching between formulations. For patients with anticipated adherence challenges, long‐acting formulations may offer therapeutic advantages due to greater tolerance to missed doses. Future studies incorporating real‐world adherence data and prospective validation would further strengthen these findings. The resulting framework provides a reproducible strategy for individualized dosing and regimen switching, addressing the clinically important challenge of uncertain efficacy caused by exposure fluctuations associated with non‐adherence.

## 4 Conclusions

This study established and qualified an integrated quantitative framework for rhGH and PEG‐rhGH in children with growth hormone deficiency, with internal and external evaluations demonstrating stable predictive performance and robust extrapolability across studies. The proposed model can predict IGF‐1 dynamics and expected growth outcomes across clinically relevant subgroups defined by age and baseline IGF‐1. In addition, an IGF‐1 SDS‐guided approach for switching from rhGH to PEG‐rhGH was established and shown to maintain comparable IGF‐1 control within accepted safety thresholds. Collectively, these results provide quantitative support for dose selection, monitoring, adherence counseling, and regimen switching in pediatric clinical practice.

## 5 Materials and Methods

### 5.1 Pharmacometric Modeling Strategy

Data from 447 subjects across four clinical trials (two phase I, one phase II, and one phase III) were included in the analysis (2005L02779 approved by the Center for Drug Evaluation of National Medical Products Administration and ClinicalTrials.gov identifiers: NCT01613573, NCT01342146, NCT01495468). The clinical datasets used for each step are summarized in Table S1 (Studies 1–4) for rhGH and Table S2 (Studies 1–2) for PEG‐rhGH (U‐PEGylated rhGH). Of note, rhGH Studies 3 and 4 correspond to the same trials as PEG‑rhGH Studies 1 and 2, in which rhGH was used as the comparator. All studies adhered to Good Clinical Practice and the Declaration of Helsinki.

A sequential modeling strategy was applied: first, a PopPK model was developed, followed by a PopPK/PD model and external validation. Then, IGF‐1‐related biomarker metrics were generated and linked to relevant clinical endpoints to support model‐informed simulations. Nonlinear mixed‑effects modeling for PopPK and PopPK/PD analyses was performed with NONMEM; model evaluation included goodness‑of‑fit plots (GOF) and visual predictive checks (VPC). Data cleaning, correlation analyses, and graphical outputs were performed using R.

The methodologies described in this section provide a concise overview. A comprehensive description of the model development process, evaluation criteria, and associated detailed methods is available in the Supporting Information Materials.

### 5.2 Population PK/PD Analysis

For rhGH, PopPK model development used dense PK data from Study 1 (healthy adults, single dose) and Study 2 (GHD children, multiple doses). Various structural models were tested, including different absorption processes (first‑order, zero‑order, or transit compartments) and elimination processes (linear, saturable, or combined). Covariate screening was performed during PopPK model building using stepwise forward inclusion (*p* < 0.05) and backward elimination (*p* < 0.001). Potentially relevant covariates (e.g., age, height, and bone age) were evaluated using linear or power models. The final PopPK model was selected based on objective function value, parameter precision, and diagnostic plots.

For PopPK/PD modeling, individual PK parameters were estimated from the final PopPK model using empirical Bayes methods and combined with PD biomarker data from Study 2. A turnover model was used to describe the relationship between drug concentration and IGF‐1 concentrations. Different PD models (linear, *E*_max_, or sigmoid *E*_max_) were tested. Additional covariate screening was performed for the PD component, including baseline IGF‐1 levels, age, bone age, and other variables. The final PopPK/PD model for rhGH was externally validated using sparse PD data from Study 3 and Study 4 (6‑month treatment) via GOF and prediction‑corrected VPC.

For PEG‐rhGH, the PopPK and PopPK/PD models were previously established in healthy participants and pediatric patients [12]. In this study, these published models were directly applied without further development. External validation was performed using data from Table S2 (Study 1 and Study 2, long‑term treatment in pediatric patients), and the established models were used for biomarker–clinical endpoint correlation analyses.

### 5.3 Biomarker‑Clinical Growth Endpoint Relationship Analysis

The biomarker–clinical growth endpoint relationship analysis used data from children with GHD: rhGH data were derived from Studies 3 and 4 in Table S1, and PEG‐rhGH data were derived from Studies 1 and 2 in Table S2 (ClinicalTrials.gov identifiers: NCT01342146, NCT01495468). Biomarker variables included IGF‐1 levels and model‐derived metrics. Efficacy endpoints were height velocity (HV), height SDS (HTSDS), and bone maturation. Exposure–efficacy relationships were initially explored via scatter plots and exploratory data analysis. Where trends were suggested, linear models were applied. A linear mixed‐effects model using all visit data was fitted for HTSDS, while cross‐sectional analyses with end‐of‐study data were conducted for HV and bone maturation.

### 5.4 Model Application

The established models were applied in two simulation scenarios using baseline parameters from a real‐world GHD cohort. Scenario 1 compared the 25‐week efficacy (HTSDS, HV) of standard daily rhGH (0.05 mg/kg/day) and weekly PEG‐rhGH (0.2 mg/kg/week) regimens. Scenario 2 quantified the impact of non‐adherence by simulating specific missed‐dose patterns. Additionally, the models were used to determine equivalent PEG‐rhGH doses for GHD children switching from common rhGH regimens, with safety verified against established IGF‐1 SDS criteria.

## Author Contributions

**Wei Wu**: conceptualization, investigation, writing – review and editing, supervision, project administration. **Chun‐Xiu Gong**: conceptualization, investigation, writing – review and editing, supervision, project administration. **Jie Mei**: conceptualization, methodology, validation, formal analysis, investigation, writing – original draft, writing – review and editing, visualization, supervision, project administration. **Ling Hou**: validation, investigation, writing – review and editing. **Yan Liang**: validation, writing – review and editing. **Yan‐qin Ying**: validation, writing – review and editing. **Feng Ye**: validation, writing – review and editing. **Ge‐Hang Ju**: conceptualization, methodology, software, validation, formal analysis, data curation, writing – original draft, visualization. **Hui‐Xiang Tian**: methodology, software, investigation, writing – original draft, writing – review and editing, visualization. **Nan Yang**: methodology, software, validation, investigation, writing – original draft, visualization. **Yun Qin**: conceptualization, writing – review and editing, supervision. **Dongsheng Ouyang**: conceptualization, writing – review and editing, resources, supervision, project administration. **Xiaoping Luo**: conceptualization, methodology, investigation, resources, writing – review and editing, supervision, project administration, funding acquisition. All authors have read and approved the final manuscript.

## Funding

Xiaoping Luo thanks the support from the National Key R&D Program of China (2023YFC2706300).

## Ethics Statement

The clinical studies (2005L02779 approved by the Center for Drug Evaluation of National Medical Products Administration and ClinicalTrials.gov identifiers: NCT01613573, NCT01342146, NCT01495468) contributing data to this analysis were conducted in accordance with the Declaration of Helsinki. Study 1 (2005L02779) was approved by the Medical Ethics Committee of the School of Pharmaceutical Sciences, Sun Yat‐sen University (Approval No. [2005] Medical Ethics Review Number 14). Studies 2–4 (NCT01613573, NCT01342146, NCT01495468) were approved by the Medical Ethics Committee of Tongji Medical College, Huazhong University of Science and Technology (approval No. [2006] Ethics Review Number (041); [2006] Ethics Review Number (020); [2007] Ethics Review Number (014)). Written informed consent was obtained from all participants or their legal guardians.

## Conflicts of Interest

The authors declare no conflicts of interest.

## Supporting information




**Methods**. Supplementary Methods.
**Table S1**: Overview of the studies included in the analysis of daily recombinant human growth hormone.
**Table S2**: Overview of the studies included in the analysis of PEGylated recombinant human growth hormone.
**Table S3**: Base and final PopPK model parameters.
**Table S4**: Base PopPK/PD model screening process.
**Table S5**: Base and final PopPK/PD parameters of rhGH.
**Table S6**: Demographic data of Phase II and III GHD validation data.
**Table S7**: Parameter estimates for short‐acting rhGH height SDS model.
**Table S8**: Parameter estimates for short‐acting rhGH growth velocity model.
**Table S9**: Parameter estimates for long‐acting PEG‐rhGH height SDS model.
**Table S10**: Parameter estimates for long‐acting PEG‐rhGH growth velocity model.
**Table S11**: Matched real‐world GHD pediatric cohort data for established biomarker‑clinical relationship and missed dose simulations.
**Table S12**: Equivalent dose switching from rhGH to PEG‐rhGH targeting IGF‐1 SDS at BIGF‐1 of 100 and 200 ng/mL.
**Table S13**: Conversion of weekly average IGF‐1 SDS to single‐day IGF‐1 SDS at steady state after weekly PEG‐rhGH administration.
**Figure S1**: Individual pharmacokinetic profiles of rhGH following a single subcutaneous administration in healthy participants.
**Figure S2**: Individual pharmacokinetic profiles of rhGH following multiple subcutaneous administrations in children with growth hormone deficiency.
**Figure S3**: Dose‐normalized C_max_ and AUC of rhGH in healthy subjects and children with growth hormone deficiency.
**Figure S4**: Individual IGF‐1 profiles of rhGH following a single subcutaneous administration in healthy participants.
**Figure S5**: Mean and individual pharmacokinetic and IGF‐1 profiles versus time following multiple rhGH dosing in children with growth hormone deficiency.
**Figure S6**: Correlation between IGF‐1 concentrations and rhGH concentrations.
**Figure S7**: Mean IGF‐1 concentrations versus mean rhGH concentrations after the first and last dosing interval.
**Figure S8**: Goodness‐of‐fit diagnostics for the base PopPK/PD model of rhGH in children with growth hormone deficiency.
**Figure S9**: Visual predictive check plot for the base PopPK/PD model of rhGH in children with growth hormone deficiency.
**Figure S10**: Individual IGF‐1 fit plots for the base PopPK/PD model of rhGH in children with growth hormone deficiency.
**Figure S11**: Correlation of inter‐individual variability for model parameters.
**Figure S12**: Individual IGF‐1 fit plots for the final PopPK/PD model of rhGH in children with growth hormone deficiency.
**Figure S13**: Goodness‐of‐fit plots for the external validation of the established PEG‐rhGH model using Phase II and III data.
**Figure S14**: Visual predictive check plots for the external validation of the established PEG‐rhGH model using Phase II and III data.
**Figure S15**: Scatter plots illustrating the correlation between efficacy variables and the change from baseline in IGF‐1 SDS following rhGH administrations.
**Figure S16**: Quartile line plots illustrating the correlation between efficacy variables and the change from baseline in IGF‐1 SDS following rhGH administrations.
**Figure S17**: Scatter plots illustrating the correlation between efficacy variables and the change from baseline in IGF‐1 SDS following PEG‐rhGH administrations.
**Figure S18**: Quartile line plots illustrating the correlation between efficacy variables and the change from baseline in IGF‐1 SDS following PEG‐rhGH administrations.
**Figure S19**: Simulated IGF‐1 SDS: rhGH 0.05 mg/kg/day to PEG‐rhGH 0.2 mg/kg/week (BIGF‐1 = 100 ng/mL).
**Figure S20**: Simulated IGF‐1 SDS: rhGH to equivalent weekly PEG‐rhGH (BIGF‐1 = 100 ng/mL).
**Figure S21**: Simulated serum drug concentrations: PEG‐rhGH 0.2 mg/kg/week to rhGH 0.05 mg/kg/day (BIGF‐1 = 50 ng/mL).
**Figure S22**: Simulated IGF‐1 SDS: PEG‐rhGH 0.2 mg/kg/week to rhGH 0.05 mg/kg/day (BIGF‐1 = 50 ng/mL).
**Figure S23**: Simulated IGF‐1 SDS: PEG‐rhGH 0.2 mg/kg/week to rhGH 0.05 mg/kg/day (BIGF‐1 = 100 ng/mL).
**Figure S24**: Simulated serum drug concentrations: PEG‐rhGH to equivalent daily rhGH (BIGF‐1 = 50 ng/mL).
**Figure S25**: Simulated IGF‐1 SDS: PEG‐rhGH to equivalent daily rhGH (BIGF‐1 = 50 ng/mL).
**Figure S26**: Simulated IGF‐1 SDS: PEG‐rhGH to equivalent daily rhGH (BIGF‐1 = 100 ng/mL).
**Figure S27**: Simulated steady‐state IGF‐1: PEG‐rhGH 0.20–0.30 mg/kg/week (BIGF‐1 = 100 ng/mL).
**Figure S28**: Simulated steady‐state IGF‐1 SDS: PEG‐rhGH 0.20–0.30 mg/kg/week (BIGF‐1 = 100 ng/mL).
**Figure S29**: Simulated steady‐state PEG‐rhGH concentrations: 0.20–0.40 mg/kg/week (BIGF‐1 = 50 ng/mL).
**Figure S30**: Simulated steady‐state IGF‐1: PEG‐rhGH 0.20–0.40 mg/kg/week (BIGF‐1 = 50 ng/mL).
**Figure S31**: Simulated steady‐state IGF‐1 SDS: PEG‐rhGH 0.20–0.40 mg/kg/week (BIGF‐1 = 50 ng/mL).
**Figure S32**: Mean difference between single‐day IGF‐1 SDS from weekly average IGF‐1 SDS at steady state after weekly PEG‐rhGH administration.

## Data Availability

The data used and/or analyzed during the current study are available from the corresponding author on reasonable request, with any data provided restricted to ensure confidentiality of all included participants.
