# Supplementary material for: Precision Dosing for Pediatric Growth Hormone Deficiency With Daily and Weekly Growth Hormone
Source: MedComm (2020). 2026 Aug 24;7(9):e70945. doi: 10.1002/mco2.70945 (PMC13501104; doi:10.1002/mco2.70945)
Supplement: Supplementary file 1 [file MCO2-7-e70945-s001.docx]

**Supplementary material**

**Precision Dosing for Pediatric Growth Hormone Deficiency With Daily and Weekly Growth Hormone**

Subtitle: Precision Dosing in Pediatric Growth Hormone Deficiency

Wei Wu^1#^, Chun-Xiu Gong^2#^, Jie Mei^3#^, Ling Hou^1^, Yan Liang^1^, Yan-qin Ying^1^, Feng Ye^1^, Ge-Hang Ju^4^, Hui-Xiang Tian^3,5^, Nan Yang^6^, Yun Qin^7*^, Dongsheng Ouyang^5*^, Xiaoping Luo^1*^

^1^Department of Pediatrics, Tongji Hospital, Tongji Medical College, Huazhong University of Science and Technology; Hubei Provincial Key Laboratory of Pediatric Genetic Metabolic and Endocrine Rare Diseases; Hubei Provincial Clinical Research Center for Children's Growth and Development and Metabolic Diseases; State Key Laboratory for Diagnosis and Treatment of Severe Zoonotic Infectious Disease, Wuhan, 430030, China.

^2^Department of Endocrinology, Genetics and Metabolism, Beijing Children’s Hospital, Capital Medical University, National Centre for Children’s Health, Beijing, 100045, China.

^3^Tianfu Jincheng Laboratory, Chengdu, 610212, China.

^4^Institute of Big Data, Central South University, Changsha, 410083, China.

^5^Department of Clinical Pharmacology, Xiangya Hospital, Central South University, Changsha, 410008, China.

^6^Center of Clinical Pharmacology, Third Xiangya Hospital, Central South University, Changsha, 410013, China.

^7^Department of Radiology, West China Hospital, Sichuan University, Chengdu, 610041, China.

^#^These authors contributed equally to this work: Wei Wu, Chun-Xiu Gong, Jie Mei.

^*^Correspondence: Xiaoping Luo ([xpluo@tjh.tjmu.edu.cn](mailto:xpluo@tjh.tjmu.edu.cn)), Dongsheng Ouyang ([801940@csu.edu.cn](mailto:801940@csu.edu.cn)), Yun Qin ([qinyun@wchscu.cn](mailto:qinyun@wchscu.cn)).

**Methods.** Supplementary Methods.

**Table S1.** Overview of the studies included in the analysis of daily recombinant human growth hormone.

**Table S2.** Overview of the studies included in the analysis of PEGylated recombinant human growth hormone.

**Table S3.** Base and final PopPK model parameters.

**Table S4.** Base PopPK/PD model screening process.

**Table S5.** Base and final PopPK/PD parameters of rhGH.

**Table S6.** Demographic data of Phase II and III GHD validation data.

**Table S7.** Parameter estimates for short‑acting rhGH height SDS model.

**Table S8.** Parameter estimates for short-acting rhGH growth velocity model.

**Table S9.** Parameter estimates for long-acting PEG-rhGH height SDS model.

**Table S10.** Parameter estimates for long-acting PEG-rhGH growth velocity model.

**Table S11.** Matched real-world GHD pediatric cohort data for established biomarker‑clinical relationship and missed dose simulations.

**Table S12.** Equivalent dose switching from rhGH to PEG-rhGH targeting IGF-1 SDS at BIGF-1 of 100 and 200 ng/mL.

**Table S13.** Conversion of weekly average IGF-1 SDS to single-day IGF-1 SDS at steady state after weekly PEG-rhGH administration.

**Figure S1.** Individual pharmacokinetic profiles of rhGH following a single subcutaneous administration in healthy participants.

**Figure S2.** Individual pharmacokinetic profiles of rhGH following multiple subcutaneous administrations in children with growth hormone deficiency.

**Figure S3.** Dose-normalized C_max_ and AUC of rhGH in healthy subjects and children with growth hormone deficiency.

**Figure S4.** Individual IGF-1 profiles of rhGH following a single subcutaneous administration in healthy participants.

**Figure S5.** Mean and individual pharmacokinetic and IGF-1 profiles versus time following multiple rhGH dosing in children with growth hormone deficiency.

**Figure S6.** Correlation between IGF-1 concentrations and rhGH concentrations.

**Figure S7.** Mean IGF-1 concentrations versus mean rhGH concentrations after the first and last dosing interval.

**Figure S8.** Goodness-of-fit diagnostics for the base PopPK/PD model of rhGH in children with growth hormone deficiency.

**Figure S9.** Visual predictive check plot for the base PopPK/PD model of rhGH in children with growth hormone deficiency.

**Figure S10.** Individual IGF-1 fit plots for the base PopPK/PD model of rhGH in children with growth hormone deficiency.

**Figure S11.** Correlation of inter-individual variability for model parameters.

**Figure S12.** Individual IGF-1 fit plots for the final PopPK/PD model of rhGH in children with growth hormone deficiency.

**Figure S13.** Goodness-of-fit plots for the external validation of the established PEG-rhGH model using Phase II and III data.

**Figure S14.** Visual predictive check plots for the external validation of the established PEG-rhGH model using Phase II and III data.

**Figure S15.** Scatter plots illustrating the correlation between efficacy variables and the change from baseline in IGF-1 SDS following rhGH administrations.

**Figure S16.** Quartile line plots illustrating the correlation between efficacy variables and the change from baseline in IGF-1 SDS following rhGH administrations.

**Figure S17.** Scatter plots illustrating the correlation between efficacy variables and the change from baseline in IGF-1 SDS following PEG-rhGH administrations.

**Figure S18.** Quartile line plots illustrating the correlation between efficacy variables and the change from baseline in IGF-1 SDS following PEG-rhGH administrations.

**Figure S19.** Simulated IGF-1 SDS: rhGH 0.05 mg/kg/day to PEG-rhGH 0.2 mg/kg/week (BIGF-1 = 100 ng/mL).

**Figure S20.** Simulated IGF-1 SDS: rhGH to equivalent weekly PEG-rhGH (BIGF-1 = 100 ng/mL).

**Figure S21.** Simulated serum drug concentrations: PEG-rhGH 0.2 mg/kg/week to rhGH 0.05 mg/kg/day (BIGF-1 = 50 ng/mL).

**Figure S22.** Simulated IGF-1 SDS: PEG-rhGH 0.2 mg/kg/week to rhGH 0.05 mg/kg/day (BIGF-1 = 50 ng/mL).

**Figure S23.** Simulated IGF-1 SDS: PEG-rhGH 0.2 mg/kg/week to rhGH 0.05 mg/kg/day (BIGF-1 = 100 ng/mL).

**Figure S24.** Simulated serum drug concentrations: PEG-rhGH to equivalent daily rhGH (BIGF-1 = 50 ng/mL).

**Figure S25.** Simulated IGF-1 SDS: PEG-rhGH to equivalent daily rhGH (BIGF-1 = 50 ng/mL).

**Figure S26.** Simulated IGF-1 SDS: PEG-rhGH to equivalent daily rhGH (BIGF-1 = 100 ng/mL).

**Figure S27.** Simulated steady-state IGF-1: PEG-rhGH 0.20–0.30 mg/kg/week (BIGF-1 = 100 ng/mL).

**Figure S28.** Simulated steady-state IGF-1 SDS: PEG-rhGH 0.20–0.30 mg/kg/week (BIGF-1 = 100 ng/mL).

**Figure S29.** Simulated steady-state PEG-rhGH concentrations: 0.20–0.40 mg/kg/week (BIGF-1 = 50 ng/mL).

**Figure S30.** Simulated steady-state IGF-1: PEG-rhGH 0.20–0.40 mg/kg/week (BIGF-1 = 50 ng/mL).

**Figure S31.** Simulated steady-state IGF-1 SDS: PEG-rhGH 0.20–0.40 mg/kg/week (BIGF-1 = 50 ng/mL).

**Figure S32.** Mean difference between single-day IGF-1 SDS and weekly average IGF-1 SDS at steady state after weekly PEG-rhGH administration.

**Supplementary Methods**

**1. Clinical data included in the analysis**

The clinical datasets used for each modeling step are summarized in Supplementary Table S1 (rhGH, Studies 1-4) and Supplementary Table S2 (PEG-rhGH, Studies 1-2). In total, data from 447 subjects across four clinical trials (two phase I, one phase II, and one phase III) were analyzed (2005L02779 approved by the Center for Drug Evaluation of National Medical Products Administration and ClinicalTrials.gov identifiers: NCT01613573, NCT01342146, NCT01495468). It should be noted that rhGH Studies 3 and 4 represent the same trials as PEG-rhGH Studies 1 and 2, with rhGH serving as the comparator in those studies. The phase I trials employed dense pharmacokinetic (PK) sampling: rhGH Study 1 (healthy adults, single dose), rhGH Study 2 (pediatric patients with GHD, multiple doses). The phase II/III trials (rhGH Studies 3-4; PEG-rhGH Studies 1-2, i.e., the same trials) were 6-month active-controlled studies with sparse pharmacodynamic (PD) sampling at weeks 0,  4, 13, and 25. The studies with the long-acting PEGylated formulation explored both weekly and biweekly dosing schedules across different patient cohorts.

All studies adhered to Good Clinical Practice and the Declaration of Helsinki. Written informed consent was obtained from all participants or their legal guardians. Study 1 (2005L02779) was approved by the the Medical Ethics Committee of the School of Pharmaceutical Sciences, Sun Yat-sen University (Approval No. [2005] Medical Ethics Review Number 14). Study 2-4 (NCT01613573, NCT01342146, NCT01495468) was approved by the Medical Ethics Committee of Tongji Medical College, Huazhong University of Science and Technology (approval No. [2006] Ethics Review Number (041); [2006] Ethics Review Number (020); [2007] Ethics Review Number (014)).

**2. Analytical methods**

Serum concentrations of both rhGH and PEG-rhGH were quantified using the same human GH ELISA platform (RayBio^®^). For rhGH, the standard sandwich format was used (anti-human GH capture antibody, biotinylated anti-human GH detection antibody). For PEG-rhGH, the detection antibody was replaced with a biotinylated anti-PEG antibody. In both assays, streptavidin-horseradish peroxidase was used as the enzyme label. Serum IGF-1 levels were analyzed using a commercially available chemiluminescence immunoassay kit.

**3. Pharmacometric modeling strategy**

Data cleaning, visualization, correlation analyses, and graphical outputs were performed using R (version 4.2.2). PopPK and PopPK/PD models were developed using nonlinear mixed-effects modeling in NONMEM^®^ (version 7.5, ICON Development Solutions, Ellicott City, MD, USA) with the first-order conditional estimation method with interaction.

A sequential modeling strategy was applied: firstly, a PopPK model was developed using dense PK data from phase I studies; secondly, empirical Bayesian estimates of individual PK parameters were used to provide drug concentrations for the subsequent PopPK/PD analysis, combined with IGF-1 data from pediatric patients to establish the PopPK/PD model; thirdly, external validation was performed using sparse PD data from phase II/III trials. Covariate screening was conducted during the PopPK and PopPK/PD steps. Subsequently, based on the final PopPK/PD models of both compounds, IGF-1 related simulated biomarker metrics were generated and correlated with height-related endpoint indicators (e.g., height velocity, height standard deviation scores) to support model-informed applications, including dose conversion, assessment of missed doses, and evaluation of high-dose scenarios.

For PEG‑rhGH, the PopPK and PopPK/PD models were previously established and published in detail. The structural forms, parameter estimates, and covariate relationships were adopted directly from the published models without further development or re‑estimation. Therefore, the following Sections 4.1–4.3 focus on the de novo model development for rhGH. For PEG‑rhGH, these published models were directly applied for external validation and further analysis using data from Study 1 and Study 2 (see Section 4.4).

**4. Population PK/PD analysis**

**4.1 Base PopPK model development**

For rhGH, dense PK data from Study 1 (healthy adults, single dose) and Study 2 (pediatric patients, multiple doses) were used to develop the PopPK model. Several candidate structural models were tested to describe the concentration-time profile, with reference to previously published models of growth hormone preparations. For absorption, first-order, zero-order, and dual absorption processes with or without lag time or transit compartments were tested. For elimination, linear, saturable, and dual elimination processes were evaluated. Baseline body weight was incorporated as an allometric scaling factor for clearance and apparent volume of distribution. The final structural model was selected based on parameter precision, objective function value (OFV), and goodness-of-fit (GOF) criteria.

**4.2 Base PopPK/PD model development**

For rhGH, individual PK parameters were estimated from the final PopPK model using empirical Bayes methods and combined with PD biomarker (IGF‑1) data from Study 2. The PD component was modeled as a turnover (indirect response) model with drug effect on the rate of IGF-1 production, as proposed previously for GH-IGF-1 axis. Different drug effect models were tested, including linear, sigmoid E_max_, and E_max_ functions with additive or proportional drug effects. To account for the observed temporal dissociation between plasma rhGH exposure and IGF-1 elevation, a transit compartment model was subsequently evaluated.

**4.3 Covariate analysis**

Covariate analysis was performed both during PopPK model building and during PopPK/PD model building for rhGH. For the PopPK model, following the selection of the structural base model, a stepwise covariate analysis was conducted on continuous covariates (including age, bone age, height, peak GH level, baseline GH level) to assess their effects on the interindividual variability of clearance and absorption rate. Peak GH level was defined as the maximum serum GH concentration observed during the diagnostic GH stimulation test before study treatment. Covariates were initially screened by visually inspecting individual empirical Bayes estimates against each candidate variable; those showing a suggestive relationship (correlation coefficient > 0.3) were retained for further testing. Additionally, correlations among covariates were also examined to assess potential collinearity. Continuous covariates were modeled using a power function centered at the median. Subsequent covariate selection employed a forward inclusion step (*p* < 0.05) followed by a stricter backward elimination step (*p* < 0.001) to avoid model overfitting.

For the PopPK/PD model, additional covariate screening was performed on PD‑related parameters. Potentially relevant covariates included baseline IGF‑1 level, age, body weight, bone age, peak GH level, baseline GH level and baseline height standard deviation score. These covariates were tested on the PD response parameters (baseline IGF‑1 production rate, E_max_, and EC₅₀) using the same stepwise procedure and statistical thresholds as described above. The final selection of PD covariates was based on changes in OFV, parameter precision, and clinical plausibility.

**4.4 Model evaluation**

Both final PopPK and PopPK/PD models for rhGH were evaluated using parameter precision, GOF plots, and OFV. Visual predictive checks (VPC) were performed to assess model predictive ability, comparing the observed data distribution with the 95% confidence intervals of simulated prediction intervals.

The external predictive performance of the established rhGH and PEG-rhGH PopPK/PD models was evaluated using both prediction-based and simulation-based verification methods, as applied to sparse PD data from Study 3 and Study 4 (6-month treatment) for rhGH and data from Study 1 and Study 2 (6-month treatment) for PEG-rhGH. Prediction-based verification involved generating GOF plots to visually compare observed values against individual or population predictions, assessing model accuracy. Simulation-based verification was performed using VPC, where the distribution of observed data was compared to the 95% confidence intervals of simulated prediction intervals. A model was considered to have adequate predictive performance if the observed median and the 5th-95th percentiles generally fell within the corresponding simulation-based confidence bands.

**5. Biomarker-clinical endpoint relationship analysis**

Data for rhGH from Studies 3 and 4 (Supplementary Table S1) and PEG-rhGH data from Studies 1 and 2 (Supplementary Table S2) were used for the biomarker-clinical growth endpoint relationship analyses in children with GHD (ClinicalTrials.gov identifiers: NCT01342146, NCT01495468). Correlation analyses between IGF-1-related metrics and clinical growth endpoints used data from children with GHD enrolled in the phase II and III trials. These analyses included rhGH or PEG-rhGH-treated patients who received ≥ 1 doses and contributed ≥ 1 measurable PD concentrations to link clinical endpoints with individual IGF-1 related metrics. Clinical growth endpoints included height standard deviation (HTSDS), height velocity(HV) and bone maturation. IGF-1-related biomarker variables selected for exploratory analysis for PEG-rhGH and rhGH were: IGF-1 and its change from baseline, IGF-1 SDS and its change from baseline, the molar ratio of IGF-1/IGFBP3 and its change from baseline, steady-state IGF-1 AUC predicted value (AUC_IGF-1_) and its change from baseline, average IGF-1 predicted value (IGF-1_avg_) and its change from baseline, average IGF-1 SDS predicted value (IGF-1_avg_ SDS) and its change from baseline, steady-state IGF-1 valley concentration value (IGF-1_min_). Among them, the first three variables are measured variables, and the last four are predictor variables obtained based on the established rhGH and PEG-rhGH PopPK/PD models.

Scatter plots of IGF-1-related biomarker variables versus clinical growth endpoints were examined, with an absolute correlation coefficient >0.3 considered suggestive of a preliminary relationship. Based on this assessment, groups were stratified by dose and exposure quartiles for exploratory data analysis, aimed at biomarker‑clinical growth endpoint relationship analysis for rhGH and PEG-rhGH. For variables showing a suggestive trend, IGF-1-related biomarker variables were formally evaluated using linear model structures, informed by prior analyses of similar PEG-rhGH analogues. Additionally, cross-sectional analyses were performed using end-of-study data for HV and bone maturation status, while a linear mixed-effects model was fitted to all available visit data for HTSDS.

**6. Model application**

**6.1 Applying the established biomarker‑clinical relationship**

Baseline characteristics (e.g., body weight, bone age) from children aged 4–10 years in a real-world GHD cohort (ClinicalTrials.gov identifier: NCT06110910) were used to parameterize the simulations. A total of 869 pediatric patients were included in this cohort, comprising 501 males (57.7%) and 368 females (42.3%). Treatment regimens were set as daily rhGH (0.05 mg/kg/day) and weekly PEG‑rhGH (0.2 mg/kg/week). To evaluate the impact of baseline IGF-1 (BIGF-1), two levels (50 and 200 ng/mL) were selected for simulation. These values were then applied to the respective population PopPK/PD and established biomarker‑clinical relationship models of rhGH and PEG‑rhGH.

Two simulation scenarios were designed. In scenario 1 (standard dosing), a 25-week treatment course was simulated for each regimen and BIGF-1 condition. Changes in HTSDS and HV were quantitatively compared between rhGH and PEG-rhGH. In scenario 2 (missed doses), steady-state simulations over seven consecutive dosing occasions were conducted to evaluate the impact of non-adherence. Three missed-dose patterns - missing 1, 2, or 3 doses (i.e., the 2nd dose; the 2nd and 3rd doses; the 2nd, 4th, and 6th doses, respectively) - were evaluated by quantifying deviations in HTSDS and HV from the normal dosing scenario for both regimens.

**6.2 Equivalent dose switching and safety evaluation**

In clinical practice, rhGH is commonly administered at 0.1 or 0.15 IU/kg/day (equivalent to 0.033 mg/kg/day and 0.05 mg/kg/day, respectively). Using the integrated PopPK/PD models, equivalent PEG-rhGH doses for switching from rhGH were determined for GHD children aged 4–10 years, targeting steady-state IGF-1 SDS as the equivalence endpoint. The safety profile of the identified equivalent and escalated doses was verified against established safety criteria, ensuring that IGF-1 SDS levels remained within the acceptable clinical range.

**Table S1.** Overview of the studies included in the analysis of daily recombinant human growth hormone.

| **No.** | **Study number** | **Study design** | **Subjects** | **Dosage regimen** | **PK sampling** | **PD sampling** | **Analysis** |
| --- | --- | --- | --- | --- | --- | --- | --- |
| 1 | 2005L02779 | A phase I clinical trial investigating the pharmacokinetics of a single dose of a PEG-rhGH | Healthy adult male subjects (n=9) | Single-dose, subcutaneous injection 0.067mg/kg. | Pre-dose (0 h) and post-dose at 0.5, 1, 1.5, 2, 3, 4, 5, 6, 8, 10, 12, 16, and 24 h. | Not applicable | PopPK analysis |
| 2 | NCT01613573 | A phase I clinical trial investigating the pharmacokinetics of multipledoses of PEG-rhGH | GHD children (n=12) | Subcutaneous injection administered once daily (QD) for 7 consecutive days. The dose was 0.0286 mg/kg/day (equivalent to 0.2 mg/kg/week). | Pre-dose (0 h) and post-dose at 1, 2, 3, 4, 6, 8, 10, 12, 16, 20, 24, 28, 48, 52, 72, 76, 96, 100, 120, 124, 144, 145, 146, 147, 148, 150, 152, 154, 156, 160, 164, 168 h. | Corresponding to pk sampling times | PopPK and PopPK/PD analysis |
| 3 | NCT01342146 | A Phase II, randomized, multicenter, open-label, parallel-group, active-controlled clinical trial (using rhGH as the control). | Children with growth hormone deficiency (n=33) | 0.25 mg/kg/week, divided into 7 doses and administered via subcutaneous injection once daily for a treatment duration of 6 months. | Not applicable | 0, 4, 13, 25 weeks | External evaluation of established popPK/PD analysis |
| 4 | NCT01495468 | A Phase III, randomized, multicenter, open-label, parallel-group, active-controlled clinical trial (using rhGH as the control). | Children with growth hormone deficiency (n=110) | 0.25 mg/kg/week, divided into 7 doses and administered via subcutaneous injection once daily for a treatment duration of 6 months. | Not applicable | 0, 4, 13, 25 weeks | External evaluation of established PopPK/PD analysis |

**Table S2.** Overview of the studies included in the analysis of PEGylated recombinant human growth hormone.

| **No.** | **Study number** | **Study design** | **Subjects** | **Dosage regimen** | **PK sampling** | **PD sampling** | **Analysis** |
| --- | --- | --- | --- | --- | --- | --- | --- |
| 1 | NCT01342146 | A Phase II, randomized, multicenter, open-label, parallel-group, active-controlled clinical trial (using rhGH as the control). | Children with growth hormone deficiency (n=61) | Low-dose group (0.1 mg/kg/week, administered once weekly, with a treatment duration of 6 months) High-dose group (0.2 mg/kg/week, administered once weekly, with a treatment duration of 6 months) | Not applicable | 0, 4, 13, 25 weeks | External evaluation of established PopPK/PD analysis |
| 2 | NCT01495468 | A Phase III, randomized, multicenter, open-label, parallel-group, active-controlled clinical trial (using rhGH as the control). | Children with growth hormone deficiency (n=222) | 0.2 mg/kg/week, administered once weekly, with a treatment duration of 6 month | Not applicable | 0, 4, 13, 25 weeks | External evaluation of established PopPK/PD analysis |

**Table S3.** Base and final PopPK model parameters.

|  | **Model with GHD children** | | **Model with healthy subjects** | | **Model with full dataset** | |
| --- | --- | --- | --- | --- | --- | --- |
| ΔOFV | 0 |  | 172.503 |  | 801.374 |  |
| Result | S, C^a^ |  | S, C |  | S, C |  |
| **Fixed-effect** | **Estimation** | **RSE**^b^ | **Estimation** | **RSE** | **Estimation** | **RSE** |
| CL, L/h | 14.2 | 18% | 16 | 22% | 14.3 | 6% |
| V1, L | 12.4 | 38% | 56.8 | 644% | 8.4 | 28% |
| KA, 1/h | 0.376 | 19% | 0.284 | 532% | 0.258 | 4% |
| ALAG1, h | 2.49 | 6% | 1.8 | 107% | 2.4 | 8% |
| D2, h | 23.1 | 2% | 2.31 | 25% | 14.4 | 3% |
| F1¹ | 0.831 | 4% | 0.539 | 462% | 0.891 | 1% |
| AGE group on F1 | / | / | / | / | 0.181 | 22% |
| **Residual error** | **Estimation** | **RSE** | **Estimation** | **RSE** | **Estimation** | **RSE** |
| Prop.error | 21.10% | 6% | 0 FIX | / | 54.49% | 5% |
| Add.error | 0.217 | 13% | 3.32 | 36% | 0.214 | 5% |
| **IIV**^d^ | **Estimation** | **RSE** | **Estimation** | **RSE** | **Estimation** | **RSE[SHR]**^c^ |
| CL, % | 11.9 | 40% [2%] | 15.5 | 178%[4%] | 13.9 | 24%[8%] |
| KA, % | / | / | / | / | 13.3 | 94%[26%] |

Abbreviation: a. S=The OFV is minimized successfully; C=The covariance matrix was calculated successfully; b. RSE: Relative standard error; c. Shrinkage; d. Inter-individual variability. CL: Clearance; V: Volume of each compartment; 1. Fraction of First-order absorption.

**Table S4.** Base PopPK/PD model screening process.

| **Run ID** | **Ref model** | **Decription** | **OFV** | **dOFV** | **Result** | **Comments** | |
| --- | --- | --- | --- | --- | --- | --- | --- |
| 061 | 059 | Indirect effect E_max_ model, mixed residual error. Parameters = (EBSL, E_max_, EC50, KOUT) | 2631.35 | / | SC, strong correlation exists between IIV on EBSL and EBSL, RSE% for IIV on EC50 is 212%, IIV on KOUT is 0.0722. | |  |
| 062 | 061 | Indirect effect E_max_ model, fixed IIV on KOUT to 0, mixed residual error. Parameters = (EBSL, E_max_, EC50, KOUT) | 2655.03 | 23.68 | SC, strong correlation exists between proportional error and EC50. | |  |
| 070 | 062 | Indirect effect E_max_ model, fixed IIV on KOUT to 0, proportional residual error. Parameters = (EBSL, E_max_, EC50, KOUT) | 2674.423 | 19.39 | SC | | Base PopPK/PD model |
| 074 | 070 | Indirect effect E_max_ model, fixed IIV on KOUT to 0, additive residual error. Parameters = (EBSL, E_max_, EC50, KOUT) | 2725.702 | 51.28 | SB, IIV on EMAX is 0.3%. | |  |
| 075 | 074 | Indirect effect E_max_ model, fixed IIV on KOUT and IIV on E_max_ to 0, additive residual error. Parameters = (EBSL, E_max_, EC50, KOUT) | 2790.352 | 64.65 | R,B | |  |
| 078 | 061 | Indirect effect sigmoid E_max_ model, mixed residual error. Parameters = (EBSL, E_max_, EC50, HILL, KOUT) | 2559.125 | -72.23 | R, IIV on HILL is 0.4%. | |  |
| 079 | 061 | Indirect effect linear model, mixed residual error. Parameters = (EBSL, SLOPE) | 2967.116 | 125.85 | T | |  |
| 081 | 078 | Indirect effect sigmoid E_max_ model, fixed IIV on HILL to 0, mixed residual error. Parameters = (EBSL, E_max_, EC50, HILL, KOUT) | 2876.338 | 317.21 | T,B | |  |

**Table S5.** Base and final PopPK/PD parameters of rhGH.

|  | **Base model** | | **Final model** | |
| --- | --- | --- | --- | --- |
| ΔOFV | 0 |  | -28.435 |  |
| Result | S, C |  | S, C |  |
| **Fixed-effect** | **Estimation** | **RSE** | **Estimation** | **RSE** |
| EBSL, μg/L | 24.9 | 19% | 30.3 | 3% |
| EMAX | 6.03 | 33% | 10.2 | 13% |
| EC50, μg/L | 0.694 | 31% | 3.4 | 53% |
| KOUT, 1/ h | 0.0327 | 50% | 0.0365 | 28% |
| BIGF1_EBSL | 1.07 | 19% | 0.96 | 5% |
| KTR | / | / | 0.623 | 7% |
| **Residual error** | **Estimation** | **RSE** | **Estimation** | **RSE** |
| Prop.error | 0.152 | 11% | 0.132 | 3% |
| Add.error | / | / | / | / |
| **IIV** | **Estimation** | **RSE[SHR]** | **Estimation** | **RSE[SHR]** |
| EMAX, % | 39.2 | 60% [0%] | / | / |
| EC50, % | / | / | 94.4 | 46%[2%] |
| KOUT, % | 32.6 | 106%[3%] | 34.8 | 53%[5%] |

Abbreviations: S, successful minimization of the objective function value; C, successful covariance step; RSE, relative standard error; IIV, interindividual variability; SHR, shrinkage; EBSL, baseline IGF-1 concentration; EMAX, maximum stimulatory effect of rhGH on IGF-1 production; EC50, rhGH concentration associated with 50% of EMAX; KOUT, first-order IGF-1 elimination rate constant; BIGF1_EBSL, effect of baseline IGF-1 on EBSL; KTR, transit rate constant.

**Table S6.** Demographic data of Phase II and III GHD validation data.

|  | **Phase II** | | | **Phase III** | |
| --- | --- | --- | --- | --- | --- |
|  | **rhGH 0.25 mg/kg/week** | **PEG-rhGH**  **0.1 mg/kg/week** | **PEG-rhGH**  **0.2 mg/kg/week** | **rhGH 0.25 mg/kg/week** | **PEG-rhGH**  **0.2 mg/kg/week** |
| n | 33 | 32 | 29 | 110 | 222 |
| Sex, n (%) |  |  |  |  |  |
| Male | 11 | 9 | 6 | 22 | 43 |
| Female | 22 | 23 | 23 | 88 | 179 |
| Age (year) | 9.82 ± 3.64 | 10.34 ± 3.3 | 10.76 ± 3.47 | 11.03 ± 3.37 | 10.64 ± 3.46 |
| Boneage (year) | 5.58 ± 2.34 | 6 ± 2.03 | 7.08 ± 2.38 | 7.2 ± 2.19 | 6.78 ± 2.24 |
| Body weight (kg) | 19.98 ± 6.03 | 20.5 ± 7.23 | 22.48 ± 6.88 | 24.49 ± 7.89 | 21.94 ± 6.22 |
| BMI (kg/m^2^) | 16.18 ± 1.96 | 16.39 ± 2.4 | 16.46 ± 2.66 | 17.01 ± 2.97 | 15.92 ± 2 |
| Height (cm) | 109.92 ± 14.51 | 110.18 ± 12.84 | 115.63 ± 11.58 | 118.44 ± 12.64 | 116.12 ± 12.62 |
| IGF-1 (ng/mL) | 64.41 ± 94.76 | 45.43 ± 53.61 | 87.62 ± 112.06 | 125.11 ± 110.21 | 102.99 ± 108.84 |
| IGF-1 SDS | -4.12 ± 2.78 | -4.5 ± 2.85 | -3.38 ± 3.78 | -2.34 ± 2.84 | -2.84 ± 3.05 |
| height velocity (cm/year) | 2.71 ± 0.91 | 2.55 ± 0.87 | 2.83 ± 0.78 | 2.27 ± 0.82 | 2.25 ± 0.86 |
| HTSDS | -0.66 ± 1.61 | -5.13 ± 1.56 | -4.65 ± 2.16 | -4.26 ± 1.9 | -4.37 ± 2.06 |

**Table S7.** Parameter estimates for short‑acting rhGH height SDS model.

| **Fixed effect** | **Estimate** | **Std. Error** | **df** | **t-value** | **p-value** |
| --- | --- | --- | --- | --- | --- |
| (Intercept) | -0.5012 | 0.236 | 419 | -2.12 | 0.0343 |
| DIGF1SDS | 0.008163 | 0.0067 | 419 | 1.22 | 0.2238 |
| WEEK | 0.03743 | 0.000999 | 419 | 37.47 | <0.0001 |
| BHTSDS | 0.9747 | 0.006702 | 137 | 145.44 | <0.0001 |
| ns(AGE,df=3)1 | -0.3703 | 0.03779 | 137 | -9.8 | <0.0001 |
| ns(AGE,df=3)2 | -0.9389 | 0.08755 | 137 | -10.72 | <0.0001 |
| ns(AGE,df=3)3 | -0.3933 | 0.0565 | 137 | -6.96 | <0.0001 |
| MPH | 0.004979 | 0.00141 | 137 | 3.53 | 0.0006 |
| DIGF1SDS:WEEK | 0.001731 | 0.00039 | 419 | 4.43 | <0.0001 |

Equation S1:

HTSDS_ij_ = − 0.5012 + 0.008163 × DIGF1SDS _ij_ + 0.03743 × WEEK _ij_ + 0.9747 × BHTSDS_ij_ − 0.3703 × ns1_ij_ − 0.9389 × ns2_ij_ − 0.3933 × ns3_ij_ + 0.004979 × MPH_i_ + 0.001731 (DIGF1SDS_ij_×WEEK_ij_) + u_i_ + ε_ij_

*HTSDS, height standard deviation; DIGF1SDS, IGF-1SDS change from baseline; BHTSDS, baseline height standard deviation score; BW, bodyweight; MPH, mid-parental height.

**Table S8.** Parameter estimates for short-acting rhGH growth velocity model.

| **Variable** | **Estimate** | **Std. Error** | **t-value** | **p-value** |
| --- | --- | --- | --- | --- |
| (Intercept) | -25.5746 | 8.9845 | -2.85 | 0.0051 |
| DIGF1SDS | 0.4953 | 0.1134 | 4.37 | <0.0001 |
| ns(AGE,df=3)1 | 3.6941 | 1.2614 | 2.93 | 0.004 |
| ns(AGE,df=3)2 | 2.9535 | 3.0145 | 0.98 | 0.329 |
| ns(AGE,df=3)3 | 0.626 | 1.4068 | 0.45 | 0.6571 |
| BONEAGE | -0.8067 | 0.1612 | -5 | <0.0001 |
| MPH | 0.2597 | 0.0569 | 4.56 | <0.0001 |
| SEX (female vs. male) | -1.8847 | 0.9276 | -2.03 | 0.0419 |

Equation S2:

HV = − 25.5746 + 0.4953 × DIGF1SDS + 3.6941 × ns1 + 2.9535 × ns2 + 0.6260 × ns3 − 0.8067 × BONEAGE + 0.2597 × MPH − 1.8847×SEX, where SEX was coded as 1 for male and 0 for female.

*HV, height velocity; DIGF1SDS, IGF-1SDS change from baseline; DOSE(0.2), dosegroup of PEG-rhGH at 0.2 mg/kg/wk; BW, bodyweight; MPH, mid-parental height.

**Table S9.** Parameter estimates for long-acting PEG-rhGH height SDS model.

| **Fixed effects** | **Estimate** | **Std. Error** | **df** | **t-value** | **p-value** |
| --- | --- | --- | --- | --- | --- |
| (Intercept) | -0.6192 | 0.1674 | 825 | -3.7 | 0.0002 |
| DIGF1SDS | 0.001848 | 0.003674 | 825 | 0.5 | 0.611 |
| WEEK | 0.03881 | 0.000774 | 825 | 50.13 | <0.0001 |
| BHTSDS | 0.9634 | 0.004819 | 273 | 199.93 | <0.0001 |
| factor(DOSE)0.2 | 0.0903 | 0.02159 | 273 | 4.18 | <0.0001 |
| ns(AGE,df=3)1 | -0.4307 | 0.02853 | 273 | -15.1 | <0.0001 |
| ns(AGE,df=3)2 | -0.8062 | 0.07994 | 273 | -10.08 | <0.0001 |
| ns(AGE,df=3)3 | -0.3616 | 0.04625 | 273 | -7.77 | <0.0001 |
| MPH | 0.004706 | 0.001003 | 273 | 4.69 | <0.0001 |
| DIGF1SDS:WEEK | 0.001492 | 0.000226 | 825 | 6.59 | <0.0001 |

**Equation S3:**

HTSDS_ij_ = −0.6192 + 0.001848 × DIGF1SDS_ij_ + 0.03881 × WEEK_ij_ + 0.9634 × BHTSDS_ij_ + 0.09030 × DOSE0.2_i_ − 0.4307 × ns1_ij_ − 0.8062 × ns2_ij_ − 0.3616 × ns3_ij_ + 0.004706 × MPH_i_ + 0.001492 × (DIGF1SDS_ij_ × WEEK_ij_) + u_i_ + ε_ij_, where u_i_~N(0,σ_u_^2^), ε_ij_~N(0,σ_ε_^2^).

*HTSDS, height standard deviation; DIGF1SDS, IGF-1SDS change from baseline; BHTSDS, baseline height standard deviation score; DOSE(0.2), dosegroup of PEG-rhGH at 0.2 mg/kg/wk; BW, bodyweight; MPH, mid-parental height.

**Table S10.** Parameter estimates for long-acting PEG-rhGH growth velocity model.

| **Variable** | **Estimate** | **Std. Error** | **t-value** | **p-value** |
| --- | --- | --- | --- | --- |
| (Intercept) | -15.1874 | 6.9123 | -2.2 | 0.0289 |
| DIGF1SDS | 0.279 | 0.07751 | 3.6 | 0.00038 |
| factor(DOSE)0.2 | 2.6978 | 0.5545 | 4.87 | <0.0001 |
| ns(AGE,df=3)1 | 4.0257 | 1.0083 | 3.99 | <0.0001 |
| ns(AGE,df=3)2 | 8.9491 | 2.4393 | 3.67 | 0.0003 |
| ns(AGE,df=3)3 | 6.0996 | 1.1232 | 5.43 | <0.0001 |
| BONEAGE | -0.6774 | 0.1611 | -4.21 | <0.0001 |
| BW | -0.1512 | 0.04582 | -3.3 | 0.0011 |
| MPH | 0.1902 | 0.04465 | 4.26 | <0.0001 |
| SEX (female vs. male) | -1.4101 | 0.8307 | -1.7 | 0.0908 |

**Equation S4:**

HV = − 15.1874 + 0.2790 × DIGF1SDS + 2.6978 × DOSE0.2 + 4.0257 × ns1 + 8.9491 × ns2 + 6.0996 × ns3 − 0.6774 × BONEAGE − 0.1512 × BW + 0.1902 × MPH − 1.4101 × SEX, where SEX was coded as 1 for male and 0 for female.

HV, height velocity; DIGF1SDS, IGF-1SDS change from baseline; DOSE(0.2), dosegroup of PEG-rhGH at 0.2 mg/kg/wk; BW, bodyweight; MPH, mid-parental height.

**Table S11.**  Matched real-world GHD pediatric cohort data for established biomarker‑clinical relationship and missed dose simulations.

| **Age** | **Sex** | **Sample size** | **Body weight^*^ (kg)** | **Bone age^*^ (year)** | **Mid-parental height^*^ (cm)** |
| --- | --- | --- | --- | --- | --- |
| 4 | Male | 165 | 14.2 | 3.10 | 156 |
| 4 | Female | 104 | 13.8 | 3.20 | 168 |
| 6 | Male | 143 | 16.6 | 4.56 | 155 |
| 6 | Female | 96 | 17.7 | 5.08 | 168 |
| 8 | Male | 110 | 22.4 | 6.65 | 155 |
| 8 | Female | 109 | 20.7 | 6.67 | 168 |
| 10 | Male | 83 | 26.2 | 8.27 | 156 |
| 10 | Female | 59 | 25.5 | 9.08 | 168 |

* Data are expressed as the mean values.

**Table S12.** Equivalent dose switching from rhGH to PEG-rhGH targeting IGF-1 SDS at BIGF-1 of 100 and 200 ng/mL.

| **Age** | **Sex** | **Dose of rhGH (mg/kg/d)** | **BIGF1 (ng/mL)** | **Equivalent dose of PEG-rhGH (mg/kg/w)** |
| --- | --- | --- | --- | --- |
| 4 | Male | 0.033 | 100 | 0.21 |
| 4 | Male | 0.05 | 100 | 0.25 |
| 4 | Female | 0.033 | 100 | 0.22 |
| 4 | Female | 0.05 | 100 | 0.26 |
| 6 | Male | 0.033 | 100 | 0.25 |
| 6 | Male | 0.05 | 100 | 0.30 |
| 6 | Female | 0.033 | 100 | 0.19 |
| 6 | Female | 0.05 | 100 | 0.22 |
| 8 | Male | 0.033 | 100 | 0.18 |
| 8 | Male | 0.05 | 100 | 0.19 |
| 8 | Female | 0.033 | 100 | 0.25 |
| 8 | Female | 0.05 | 100 | 0.28 |
| 10 | Male | 0.033 | 100 | 0.16 |
| 10 | Male | 0.05 | 100 | 0.18 |
| 10 | Female | 0.033 | 100 | 0.22 |
| 10 | Female | 0.05 | 100 | 0.25 |
| 4 | Male | 0.033 | 200 | 0.20 |
| 4 | Male | 0.05 | 200 | 0.23 |
| 4 | Female | 0.033 | 200 | 0.22 |
| 4 | Female | 0.05 | 200 | 0.26 |
| 6 | Male | 0.033 | 200 | 0.25 |
| 6 | Male | 0.05 | 200 | 0.30 |

(Continues)

Table S12 (Continued)

| **Age** | **Sex** | **Dose of rhGH (mg/kg/d)** | **BIGF1 (ng/mL)** | **Equivalent dose of PEG-rhGH (mg/kg/w)** |
| --- | --- | --- | --- | --- |
| 6 | Female | 0.033 | 200 | 0.19 |
| 6 | Female | 0.05 | 200 | 0.22 |
| 8 | Male | 0.033 | 200 | 0.17 |
| 8 | Male | 0.05 | 200 | 0.19 |
| 8 | Female | 0.033 | 200 | 0.23 |
| 8 | Female | 0.05 | 200 | 0.28 |
| 10 | Male | 0.033 | 200 | 0.16 |
| 10 | Male | 0.05 | 200 | 0.18 |
| 10 | Female | 0.033 | 200 | 0.22 |
| 10 | Female | 0.05 | 200 | 0.25 |

**Table S13.** Conversion of weekly average IGF-1 SDS to single-day IGF-1 SDS at steady state after weekly PEG-rhGH administration.

| **Day** | **Mean difference (Day-avg)** | **90% PI** | **R^2^** |
| --- | --- | --- | --- |
| 1 | +0.23 | [-0.61, 1.19] | 0.95 |
| 2 | +0.70 | [-0.34, 1.89] | 0.92 |
| 3 | +0.69 | [-0.27, 1.80] | 0.93 |
| 4 | +0.40 | [-0.44, 1.30] | 0.95 |
| 5 | -0.13 | [-1.00, 0.67] | 0.96 |
| 6 | -0.70 | [-1.78, 0.19] | 0.95 |
| 7 | -1.20 | [-2.55, -0.13] | 0.91 |

Abbreviations: avg, weekly average IGF-1 SDS; PI, prediction interval; Mean difference (Day-avg) represents single-day IGF-1 SDS minus weekly average IGF-1 SDS. Therefore, single-day IGF-1 SDS can be estimated as weekly average IGF-1 SDS + mean difference (Day-avg).

**Figure S1.** Individual pharmacokinetic profiles of rhGH following a single subcutaneous administration in healthy participants.

**Figure S2.** Individual pharmacokinetic profiles of rhGH following multiple subcutaneous administrations in children with growth hormone deficiency.

**Figure S3.** Dose-normalized Cmax and AUC of rhGH in healthy subjects and children with growth hormone deficiency. The left and right panels show dose-normalized Cmax and AUC, respectively. In each panel, data are grouped by dosing interval: GHD children at the first interval (dark blue), GHD children at the last interval (medium blue), and healthy subjects after a single dose (light blue).

**Figure S4.** Individual IGF-1 profiles following multiple subcutaneous administrations of rhGH in children with GHD.

**Figure S5.** Mean and individual pharmacokinetic and IGF-1 profiles versus time following multiple rhGH dosing in children with growth hormone deficiency. The left y-axis represents rhGH concentrations, and the right y-axis represents IGF-1 concentrations. Individual observations are shown as scatter points overlaid on the mean profile (solid line).

**Figure S6.** Correlation between IGF-1 concentrations and rhGH concentrations. Individual observations are shown as open black circles. The blue line represents the loess-smoothed trend, with the light-blue shaded area indicating the corresponding 95% confidence interval.

**Figure S7.** Mean IGF-1 concentrations versus mean rhGH concentrations after the first and last dosing interval. The blue lines and solid circles correspond to the first interval (0-24 h), while the pink line and solid circles correspond to the last interval (144-168 h).

**Figure S8.** Goodness-of-fit diagnostics for the base PopPK/PD model of rhGH in children with growth hormone deficiency. In all panels, observed IGF-1 concentrations are shown as blue circles, the identity line as a black solid line, and a loess smooth trend as a green dashed line. The panels depict, from top left to bottom right: individual predicted versus observed concentrations, population predicted versus observed concentrations, conditional weighted residuals (CWRES) versus time, and CWRES versus population predicted concentrations.

**Figure S9.** Visual predictive check plot for the base PopPK/PD model of rhGH in children with growth hormone deficiency. The observed concentrations are represented by green solid circles. The solid blue line indicates the 50th percentile of the observed data, while the solid red lines mark the 5th and 95th percentiles. The blue shaded area shows the 90% prediction interval for the 50th percentile of the simulated concentrations, and the red shaded areas represent the corresponding 90% prediction intervals for the 5th and 95th percentiles of the simulated concentrations.

**Figure S10.** Individual IGF-1 fit plots for the base PopPK/PD model of rhGH in children with growth hormone deficiency. Observed IGF-1 concentrations are shown as pink open circles. The pink dashed line represents population IGF-1 predictions, and the pink solid line represents individual IGF-1 predictions.

**Figure S11.** Correlation of inter-individual variability for model parameters. ETAEBSL, ETAEMAX, and ETAEC50 denote the inter-individual variability (IIV) of the baseline effect, maximum effect, and estimated time of arrival to EC50, respectively. BW represents body weight; HSD, baseline height standard deviation. PGH and BGH are defined as the peak and baseline growth hormone levels from the stimulation test, respectively. BIGF1 represents the baseline IGF-1 level. A star marker in the blue area denotes an absolute correlation coefficient > 0.3 between a covariate and a parameter. A star marker in the pink area denotes an absolute correlation coefficient > 0.8 between two covariates.

**Figure S12.** Individual IGF-1 fit plots for the final PopPK/PD model of rhGH in children with growth hormone deficiency. Observed IGF-1 concentrations are shown as pink open circles. The pink dashed line represents population IGF-1 predictions, and the pink solid line represents individual IGF-1 predictions.

**Figure S13.** Goodness-of-fit plots for the external validation of the established PEG-rhGH model using Phase II and III data. In all panels, observed IGF-1 concentrations of Phase II and phase III studies are reprensented by pink and blue circles, respectively; the identity line is shown as a solid black line, and a LOESS smooth trend is indicated by a green dashed line. The panels (arranged from top left to bottom right) displayed the following: individual predicted versus observed concentrations, population predicted versus observed concentrations, conditional weighted residuals (CWRES) versus time, and CWRES versus population predicted concentrations.

**Figure S14.** Visual predictive check plots for the external validation of the established PEG-rhGH model using Phase II and III data. The left and right panels display the VPC for the Phase II and Phase III evaluation results, respectively. In all pannels, the observed concentrations are represented by green solid circles. The solid blue line indicates the 50th percentile of the observed data, while the solid red lines mark the 5th and 95th percentiles. The blue shaded area shows the 90% prediction interval for the 50th percentile of the simulated concentrations, and the red shaded areas represent the corresponding 90% prediction intervals for the 5th and 95th percentiles of the simulated concentrations.

**Figure S15.** Scatter plots illustrating the correlation between efficacy variables and the change from baseline in IGF-1 SDS following rhGH administrations. From left to right, the panels correspond to the following exposure metrics: change from baseline in height standard deviation (SDS) across all visit weeks, height velocity at week 25, and bone maturation at week 25. In all panels, R values denote the Pearson correlation coefficient, and the p values indicate the corresponding result of the statistical test. In the leftmost panel, data points are color-coded by assessment time point: pink (week 4), green (week 13), and blue (week 25) circles represent the change from baseline in height SDS at the respective visits.

**Figure S16.** Quartile line plots illustrating the correlation between efficacy variables and the change from baseline in IGF-1 SDS following rhGH administrations. From left to right, the panel present analyses for the following exposure metrics: change from baseline in height standard deviation (SDS) across all visit weeks, height velocity at week 25, and bone maturation at week 25. In all panels, the data are stratified into quartiles (Q1~Q4) based on the corresponding changes from baseline IGF-1 SDS, and are color-coded as follows: pink (Q1), green (Q2), blue (Q3), and purple (Q4).

**Figure S17.** Scatter plots illustrating the correlation between efficacy variables and the change from baseline in IGF-1 SDS following PEG-rhGH administrations. From left to right, the panels correspond to the following exposure metrics: change from baseline in height standard deviation (SDS) across all visit weeks, height velocity at week 25, and bone maturation at week 25. In all panels, R values denote the Pearson correlation coefficient, and the p values indicate the corresponding result of the statistical test. In the leftmost panel, data points are color-coded by assessment time point: pink (week 4), green (week 13), and blue (week 25) circles represent the change from baseline in height SDS at the respective visits.

**Figure S18.** Quartile line plots illustrating the correlation between efficacy variables and the change from baseline in IGF-1 SDS following PEG-rhGH administrations. From left to right, the panel present analyses for the following exposure metrics: change from baseline in height standard deviation (SDS) across all visit weeks, height velocity at week 25, and bone maturation at week 25. In all panels, the data are stratified into quartiles (Q1~Q4) based on the corresponding changes from baseline IGF-1 SDS, and are color-coded as follows: pink (Q1), green (Q2), blue (Q3), and purple (Q4).

**Figure S19.** Simulated IGF-1 SDS: rhGH 0.05 mg/kg/day to PEG-rhGH 0.2 mg/kg/week (BIGF-1 = 100 ng/mL). This simulation was used to evaluate IGF-1 SDS continuity after switching from daily rhGH to the labeled weekly PEG-rhGH dose. Blue and red solid lines represent model-predicted median IGF-1 SDS values during rhGH and PEG-rhGH treatment periods, respectively. Shaded ribbons indicate the 90% prediction intervals (5th–95th percentiles). Vertical dashed lines denote the time of treatment switch. Horizontal dashed lines indicate reference thresholds at 0 and +2 SDS.

**Figure S20.**  Simulated IGF-1 SDS: rhGH to equivalent weekly PEG-rhGH (BIGF-1 = 100 ng/mL). This simulation was used to assess whether model-derived equivalent PEG-rhGH doses better preserved IGF-1 SDS after switching. The same graphical conventions as in Figure S19 are used. Equivalent PEG-rhGH doses were selected to target comparable IGF-1 SDS exposure after treatment switch.

**Figure S21.** Simulated serum drug concentrations: PEG-rhGH 0.2 mg/kg/week to rhGH 0.05 mg/kg/day (BIGF-1 = 50 ng/mL). This simulation was used to explore reverse switching from PEG-rhGH to rhGH and to characterize the influence of the prolonged PEG-rhGH pharmacokinetic tail on subsequent drug concentration profiles. Blue and red solid lines represent model-predicted median concentration values during rhGH and PEG-rhGH treatment periods, respectively. Shaded ribbons indicate the 90% prediction intervals (5th–95th percentiles). Vertical dashed lines denote the time of treatment switch.

**Figure S22.** Simulated IGF-1 SDS: PEG-rhGH 0.2 mg/kg/week to rhGH 0.05 mg/kg/day (BIGF-1 = 50 ng/mL). This simulation was used to evaluate the corresponding IGF-1 SDS response after reverse switching from PEG-rhGH to fixed-dose daily rhGH. Blue and red solid lines represent model-predicted median IGF-1 SDS values during rhGH and PEG-rhGH treatment periods, respectively. Shaded ribbons indicate the 90% prediction intervals (5th–95th percentiles). Vertical dashed lines denote the time of treatment switch. Horizontal dashed lines indicate reference thresholds at 0 and +2 SDS.

**Figure S23.** Simulated IGF-1 SDS: PEG-rhGH 0.2 mg/kg/week to rhGH 0.05 mg/kg/day (BIGF-1 = 100 ng/mL). This simulation extends the fixed-dose reverse-switching scenario to a higher BIGF-1 level. The same graphical conventions as in Figure S22 are used. Dashed horizontal lines indicate reference thresholds at 0, +2, and +3 SDS.

**Figure S24.** Simulated serum drug concentrations: PEG-rhGH to equivalent daily rhGH (BIGF-1 = 50 ng/mL). This simulation was used to explore reverse switching using model-derived equivalent daily rhGH doses. The same graphical conventions as in Figure S21 are used. Equivalent rhGH doses were selected to target comparable IGF-1 SDS exposure after treatment switch.

**Figure S25.** Simulated IGF-1 SDS: PEG-rhGH to equivalent daily rhGH (BIGF-1 = 50 ng/mL). This simulation was used to evaluate the corresponding IGF-1 SDS response after reverse switching to model-derived equivalent daily rhGH doses. The same graphical conventions as in Figure S22 are used. Horizontal dashed lines indicate reference thresholds at 0, +2, and +3 SDS.

**Figure S26.** Simulated IGF-1 SDS: PEG-rhGH to equivalent daily rhGH (BIGF-1 = 100 ng/mL). This simulation extends the equivalent-dose reverse-switching scenario to a higher BIGF-1 level. The same graphical conventions as in Figure S22 are used. Dashed horizontal lines indicate reference thresholds at 0, +2, and +3 SDS.

**Figure S27.** Simulated steady-state IGF-1: PEG-rhGH 0.20–0.30 mg/kg/week (BIGF-1 = 100 ng/mL). This simulation was used to assess IGF-1 concentration profiles across the proposed equivalent PEG-rhGH dose range at a higher BIGF-1 level. Curves represent model-predicted median IGF-1 concentration levels during the final weekly dosing interval (Week 7, 1008–1176 h). Different blue lines indicate different PEG-rhGH dose levels.

**Figure S28.** Simulated steady-state IGF-1 SDS: PEG-rhGH 0.20–0.30 mg/kg/week (BIGF-1 = 100 ng/mL). This simulation was used to evaluate whether the proposed equivalent PEG-rhGH dose range maintained IGF-1 SDS within clinically relevant monitoring thresholds at a higher BIGF-1 level. The same dose-level color conventions as in Figure S27 are used. Curves represent model-predicted median IGF-1 SDS values during the final weekly dosing interval (Week 7, 1008–1176 h). Dashed horizontal lines indicate reference thresholds at 0, +2, and +3 SDS.

**Figure S29.** Simulated steady-state PEG-rhGH concentrations: 0.20–0.40 mg/kg/week (BIGF-1 = 50 ng/mL). This simulation was used to characterize PEG-rhGH concentration profiles across an extended dose range beyond the proposed equivalent dose range. Curves represent model-predicted median PEG-rhGH concentration levels during the final weekly dosing interval (Week 7, 1008–1176 h). Different blue lines indicate different PEG-rhGH dose levels.

**Figure S30.** Simulated steady-state IGF-1: PEG-rhGH 0.20–0.40 mg/kg/week (BIGF-1 = 50 ng/mL). This simulation was used to characterize IGF-1 concentration profiles across the extended PEG-rhGH dose range. The same dose-level color conventions as in Figure S29 are used. Curves represent model-predicted median IGF-1 concentration levels during the final weekly dosing interval (Week 7, 1008–1176 h).

**Figure S31.**  Simulated steady-state IGF-1 SDS: PEG-rhGH 0.20–0.40 mg/kg/week (BIGF-1 = 50 ng/mL). This simulation was used to evaluate the IGF-1 SDS safety margin across the extended PEG-rhGH dose range. The same dose-level color conventions as in Figure S29 are used. Curves represent model-predicted median IGF-1 SDS values during the final weekly dosing interval (Week 7, 1008–1176 h). Dashed horizontal lines indicate reference thresholds at 0, +2, and +3 SDS.

**Figure S32.** Mean difference between single-day IGF-1 SDS and weekly average IGF-1 SDS at steady state after weekly PEG-rhGH administration. This analysis was used to support practical IGF-1 SDS monitoring by identifying which post-dose sampling day most closely approximated the weekly average IGF-1 SDS. Points and curves represent the model-predicted mean difference between single-day IGF-1 SDS and weekly average IGF-1 SDS during the final weekly dosing interval at steady state. The dashed horizontal line represents the weekly average IGF-1 SDS, corresponding to zero difference.
